# Informed decision-making among students analyzing their personal genomes on a whole genome sequencing course: a longitudinal cohort study

**DOI:** 10.1186/gm518

**Published:** 2013-12-30

**Authors:** Saskia C Sanderson, Michael D Linderman, Andrew Kasarskis, Ali Bashir, George A Diaz, Milind C Mahajan, Hardik Shah, Melissa Wasserstein, Randi E Zinberg, Micol Zweig, Eric E Schadt

**Affiliations:** 1 Department of Genetics and Genomic Sciences, Icahn School of Medicine at Mount Sinai, 1425 Madison Avenue, New York, NY 10029, USA; 2 Icahn Institute for Genomics and Multiscale Biology, Icahn School of Medicine at Mount Sinai, 1425 Madison Avenue, New York, NY 10029, USA

## Abstract

**Background:**

Multiple laboratories now offer clinical whole genome sequencing (WGS). We anticipate WGS becoming routinely used in research and clinical practice. Many institutions are exploring how best to educate geneticists and other professionals about WGS. Providing students in WGS courses with the option to analyze their own genome sequence is one strategy that might enhance students’ engagement and motivation to learn about personal genomics. However, if this option is presented to students, it is vital they make informed decisions, do not feel pressured into analyzing their own genomes by their course directors or peers, and feel free to analyze a third-party genome if they prefer. We therefore developed a 26-hour introductory genomics course in part to help students make informed decisions about whether to receive personal WGS data in a subsequent advanced genomics course. In the advanced course, they had the option to receive their own personal genome data, or an anonymous genome, at no financial cost to them. Our primary aims were to examine whether students made informed decisions regarding analyzing their personal genomes, and whether there was evidence that the introductory course enabled the students to make a more informed decision.

**Methods:**

This was a longitudinal cohort study in which students (*N* = 19) completed questionnaires assessing their intentions, informed decision-making, attitudes and knowledge before (T1) and after (T2) the introductory course, and before the advanced course (T3). Informed decision-making was assessed using the Decisional Conflict Scale.

**Results:**

At the start of the introductory course (T1), most (17/19) students intended to receive their personal WGS data in the subsequent course, but many expressed conflict around this decision. Decisional conflict decreased after the introductory course (T2) indicating there was an increase in informed decision-making, and did not change before the advanced course (T3). This suggests that it was the introductory course content rather than simply time passing that had the effect. In the advanced course, all (19/19) students opted to receive their personal WGS data. No changes in technical knowledge of genomics were observed. Overall attitudes towards WGS were broadly positive.

**Conclusions:**

Providing students with intensive introductory education about WGS may help them make informed decisions about whether or not to work with their personal WGS data in an educational setting.

## Background

### Whole genome sequencing inside and outside the clinic

As the technology advances and costs fall, whole genome sequencing (WGS) – the sequencing of all or most of the DNA in the genome of an individual – is projected to replace targeted genetic tests and single nucleotide polymorphism (SNP) microarrays in the future. Many laboratories already offer WGS for clinical and research purposes. The deluge of genomic information produced by WGS brings with it a multitude of both opportunities and challenges in a clinical setting: the opportunities include improved diagnoses, disease treatment and prevention; the challenges include uncertainties regarding what to do with incidental or secondary findings that may arise, how to decide what to do with variants of uncertain significance and the extent to which patients’ preferences should be taken into account regarding what results should be returned. The advances in genomics are outpacing ‘unprepared professionals’ in the medical arena and pose challenges to health-care providers regarding their current ability to understand genomics data, appropriately translate them into actionable results and convey such results to patients [1].

A further consideration is that genetic testing and sequencing are no longer restricted to clinic settings. Although personal genomics companies have yet to expand to WGS, personal genomics companies such as 23andMe provide extensive personal microarray-based genomic data and interpretations of that data to consumers, and have provided whole exome sequencing data and results to a subset of their consumers. As a consequence, patients may already be gaining a more direct understanding of their disease risk, disease subtypes and sensitivities to different medicines based on their genomic information without the involvement of a clinician. Although consumer genomics is undoubtedly in a state of flux at the present time (the Food and Drug Administration (FDA) recently ordered 23andMe to cease marketing their existing product [2]), the FDA Commissioner recently wrote that ‘access to tests through a direct-to-consumer model will allow consumers to take a more active role in certain aspects of their health’ [3], suggesting that personal genomic information may be more widely available in the future. Consumers can be viewed as being their own best advocates for their well-being, and many may wish to access more genomic and other personal information outside of a clinic and seek better understanding of their conditions implied by these data. Medical professionals will need to keep pace with these developments in personal genomics [1], whether the personal genomic information is obtained inside or outside of a clinical setting.

### Training medical professionals: genomics courses using commercial personal genomics services

Advanced training is needed to help genetics professionals, and clinicians more generally, understand large and complex datasets such as those produced by WGS. Many institutions of higher learning are now implementing advanced genomics courses to respond to this need. Some institutions are exploring the use of novel, interactive and engaging approaches to replace purely didactic lectures [4], including the use of simulators or standardized patients, and internet-based genomics education for physicians [4]. Another potential approach that has been considered and debated is to engage health-care students by using their own personal genomic data [5,6]. This is based on the premise that students who engage in ‘self-testing’ and receive personal genomic information may benefit more from the training than students who view the genomic information of an anonymous third party [5,7-9] cf. [10]. While much of this is optimistic speculation and debate focused on ethics rather than actual implementation (one institution proposed personal genetic testing of students but, after considerable institutional deliberation, ultimately opted to use aggregate genetic information instead [6]), a handful of institutions of higher learning have begun offering courses in which students have the option of working with their own personal genomic data. There is precedent for this in other areas of medical training in that students often perform medical exams on themselves and obtain personal health-related information about themselves using a test they are learning about [1]. Yet there is currently much rigorous debate on whether genomics courses in the educational setting should include the option of allowing students to use their own genomic data, and concerns about the ethics of pursuing this path [10-12].

Empirical data to inform this debate is urgently needed. Despite several institutions offering personal genomic testing within an educational setting to their students, few have published empirical evidence regarding the students’ experiences of these courses. Only two published studies have reported on students’ experiences of accessing their own genomic data in the classroom [12,13]. The first of these studies [12] assessed the experiences of students on the elective GENE 210 ‘Genomics and personalized medicine’ course offered at Stanford University School of Medicine in the summer of 2010, in which students could obtain microarray-based genetic testing from 23andMe and Navigenics at a discounted rate, which included common disease risk prediction, drug metabolism and ancestry [12]. In this study, qualitative interviews were conducted at three time points with 10 of the 46 students enrolled on the course. The results from these interviews suggested that the students were skeptical of some of their genomic test results, but that they found the experience generally beneficial. Of note, few students recalled the informed consent details, and few took up the offered genetic counseling [12]. In the second, quantitative questionnaire study [13], the Stanford group found that out of 31 students given the option of using their own commercially produced genetic test results in the classroom, the 23 students who opted to do so showed significant increases in technical knowledge of genomics, whereas the eight students who opted to use third-party results did not, tentatively suggesting that using personal genomic information in the classroom increases learning.

### Moving from receiving commercially produced genomic test results to handling, analyzing and interpreting an entire genome sequence

Courses such as that at Stanford are beginning to address how biomedical professionals and students may respond to receiving their own results from genomic testing. However, students in the courses that use commercial genomic tests do not directly engage the full complexity of WGS data. They have little or no hands-on experience of detecting, analyzing and interpreting DNA variants to determine, for example, whether or not they are of clinical significance. Current courses that rely on consumer genomics products and test results also restrict attention to a fraction of the DNA variation that exists in any individual, largely ignoring rarer variants that may play more important roles in an individual’s disease risk or other traits.

Given that WGS provides the complete set of inherited genetic information on a person, it is reasonable to expect that WGS will eventually become the standard in assessing genomic variation in health care and other settings in the future. It is therefore timely to explore the implications of moving from providing students with personal genomic test results already analyzed and interpreted by commercial entities to providing students with access to their own, personal, entire WGS dataset in all its scope and complexity. Students who work with their own personal genomes while learning about WGS may be more engaged with the process, more motivated to learn about WGS, spend more time gaining the skills to analyze and interpret their personal genome data and appreciate more directly the impact of the testing, decision-making and subsequent ethical, social and familial implications than if they use that of an anonymous third party. However, there are many vital ethical and practical questions that need to be addressed before this practice can or should be implemented on a wide scale.

### The importance of informed decision-making

Without wishing to invoke ‘genetic exceptionalism’ (the view that genetic or genomic information is special and that genetic and genomic tests must therefore be treated differently and more strictly regulated than other types of medical information [14]), a valid ethical and pragmatic question that has not yet been tackled empirically is whether students are capable of making independent, informed choices or ‘informed decisions’ about whether or not to receive their personal genome sequence, which they may analyze and interpret as part of their genomics training. The study of informed decision-making is related to but distinct from that of informed consent. Informed decision-making can be defined as a cognitive and emotional process that leads to a decision being made and subsequent action being carried out, based on sufficient understanding and awareness of the risks, benefits, limitations, uncertainties and alternatives regarding the technology in question, and that is consistent with the individual’s attitudes or views [15,16]. The concept of informed consent in the research context similarly includes a focus on whether individuals have sufficient understanding on which to base their decisions, but is more tightly tied to how that consent is obtained and documented, and to key sets of research ethics principles such as the Nuremberg Code (for an in-depth conceptual and historical review of informed consent see [17]). While there is also a rich and thoughtful literature on the ethics, practical considerations and challenges inherent in obtaining informed consent specifically within the personal genomics arena (see, for example, [18,19]), this is largely outside the scope of the present report. Here, we focus primarily on providing empirical data on the psychological underpinnings of informed decision-making from the students’ perspectives, albeit with a view to informing the ongoing debates about consent and ethics in personal genomics.

In the educational setting, there are particular concerns that students may not be able to make informed, personal choices about whether or not to receive personal genomic data as part of their genomics training because they will feel pressured to analyze their personal genomes, either directly or indirectly by their professors or peers, even if this goes against their wishes. Currently, there is no empirical data to support or refute these concerns. Empirical evidence addressing whether students are making independent, informed decisions about analyzing their own personal genomes, and whether efforts to help students make informed decisions are successful, would shed valuable light on a major ethical concern surrounding personal genomes in the classroom.

One useful conceptual framework for the empirical examination of informed decision-making is derived from the construct of decisional conflict: decision uncertainty or ‘decisional conflict’ is ‘a state of uncertainty about the course of action to take’ and is ‘likely when making choices involving risk or uncertainty of outcomes, high stakes in terms of potential gains and losses, the need to make value tradeoffs in selecting a course of action, and anticipated regret over the positive aspects of rejected options’ [20]. In this model, factors hypothesized to contribute to an individual’s decisional conflict include lack of information, unclear values, skill deficits in making or implementing decisions, emotional distress and critically ‘perceived pressures from important others who are imposing their views.’ O’Connor’s Decisional Conflict Scale (DCS) [20] was developed to assess decision uncertainty particularly with a focus on the effects of informed choice interventions, on the premise that measured reductions in decision uncertainty may be viewed as indicators of informed choice interventions being successful. The DCS provides a useful practical measurement tool for use in empirical research on informed decision-making in personal genomics.

### The present study

With the exception of the two studies published by the Stanford group [12,13], no other empirical data regarding students’ actual experiences of obtaining personal genomic data in an educational setting is yet available, either regarding the downstream psychological and educational effects or the upstream issues around informed decision-making. Moreover, no group has yet explored the issues and potential gains to be had by having students analyze their own entire complex WGS datasets rather than handing students already interpreted genomic results. Yet such data will be vital to efforts to determine the cost-benefit ratio of incorporating personal genomics into future educational efforts as costs of sequencing decline.

We therefore set out to explore student views on the use of personal genomics in a two-part genomics course in which students had the option to access, analyze and interpret their own, personal, entire WGS datasets for themselves. We were aware that this was the first time any educational institution had enabled an entire class of students to analyze their own personal genomes, and so adopted a ‘first do no harm’ position with the course. We exercised great care and caution and placed a major focus on carrying out this study in an ethically responsible way. We deliberately engaged the students in an intensive two and a half week introductory course (26 hours in total) over the summer of 2012 in an effort to ensure the students had ample information on which to base their decisions about moving forward with personal genome sequencing as part of the subsequent advanced course in the fall of 2012, in which they had the option to access, analyze, interpret and keep their own personal genome sequence data. The students also had one and a half months during which to consider their decision before they had blood drawn for WGS, and many support resources were made available to them including genetic counselors both within and outside the institution, and access to the student mental health services.

Our overarching primary aims in this project were twofold. First, we aimed to determine whether the introductory course successfully achieved the goal of helping the students make more informed decisions about whether to proceed with personal genome sequencing for educational purposes in the advanced course. Second, we aimed to explore the educational and psychological impact of the students having had the option of analyzing their personal, entire genome sequence data as part of their advanced genomics training. We also explored the students’ attitudes towards WGS more broadly, who they discussed their decision and personal WGS-based results with, and how their views and understanding changed over time. Our overarching conceptual framework for the study is shown in (Additional file 1: Figure S1) [15,16]. In the present paper, we present the results from the first part of the study, that is, the part focused on informed decision-making upstream in the process. Our specific aims here were:

#### Aim 1

To describe the baseline attitudes, knowledge, anticipated decisions and degree of informed decision-making regarding obtaining personal WGS in an educational setting, among students at the start of a two-part introductory and advanced genomics course (T1).

#### Aim 2

To examine whether the students’ attitudes, knowledge, anticipated decisions and degree of informed decision-making regarding obtaining personal WGS in an educational setting changed over time between the start and end of the introductory genomics course (T1 to T2).

#### Aim 3

To examine whether the students’ attitudes, knowledge, anticipated decisions and degree of informed decision-making regarding obtaining personal WGS in an educational setting changed over time between the end of the introductory and start of the advanced genomics course (T2 to T3), and who the students had discussed their decision with at T3. We also report the proportion of students who opted to analyze their own versus a third-party genome after T3.

We hypothesized that this self-selected group of students would express positive attitudes towards WGS generally and WGS in an educational context specifically, and considerable interest in having their own genomes sequenced, but that their knowledge would be low, when they started the introductory course at baseline (T1). We also hypothesized that their knowledge would increase between baseline and the end of the introductory course, that their attitudes would become less positive as they learned more about the risks, limitations and uncertainties surrounding WGS technology during the introductory course and that their interest in having their own genomes sequenced would also consequently wane, but that they would feel they were making significantly more informed choices by the end compared to the start of the introductory course (T1 to T2). Finally, we hypothesized that few changes in attitudes, knowledge or interest would be observed between the end of the introductory course and start of the advanced course (T2 to T3). By collecting and comparing data at all three time points (T1, T2 and T3), the study design allowed us to explore whether greater changes were observed in response to the introductory course (that is, between the start and end of the first course, T1 to T2) than might reasonably have been expected simply due to time passing (indicated by the time passing between the end of the first course and the start of the second course, T2 to T3). For completeness and to see whether improvements in informed decision variables were maintained, we also compared responses between the start of the introductory and the start of the advanced courses (T1 to T3). In the present article, we report the results from analyses in which we assessed whether the students made informed decisions, and whether their decisions appeared to be more informed as a consequence of having engaged in the introductory course. We also report the students’ attitudes towards WGS more broadly and who they discussed their decision with.

## Methods

### Study design and procedure

This was a longitudinal cohort study of students enrolled in a two-part introductory and advanced genomics course at the Icahn School of Medicine at Mount Sinai (ISMMS), New York. The introductory course, ‘Introduction to human genome sequencing’, was offered in July and August 2012, and the advanced course, ‘Practical analysis of your personal genome’, was offered in the fall semester of 2012. Students completed questionnaires about a range of topics including their attitudes towards the use of personal genomes in the educational setting before and after each course (see Additional file 2: Figure S2 for a participant flow chart). Through the use of the surveys we attempted to engage the students in the decision-making process about incorporating personal genomes into a genome-sequencing course, and collect input on those factors that had the potential to help or hinder the process. Student views presented in this article were assessed via three paper questionnaire surveys, one before and after the introductory course (see Additional file 3 for the first questionnaire) and one before the advanced course (see Additional file 4 for the second questionnaire). See Additional file 5: Table S1 for an overview of all the data collection time points in this study and the measures assessed at each time point.

The course and research study were developed by a multidisciplinary group of experts in genetic counselling, medical genetics, health psychology and bioinformatics. The aim of the introductory genomics course, ‘Introduction to human genome sequencing’, was to provide the genomic, computing and algorithm fundamentals required to assemble, analyze and interpret WGS data, and appreciation of the potential risks and benefits and the ethical and psychological issues that could be raised (see Additional file 6 for the introductory course syllabus). During this course, students were also informed that when examining their WGS data during the advanced course, they could exclude data they did not wish to see. This course was a prerequisite for the advanced genomics course. The advanced genomics course, ‘Practical analysis of your personal genome’, was developed with the aim of providing students with the skills to analyze WGS data, and to carry out a variety of interpretations on these analysis results, including general assessments of genome-wide small nucleotide variations, identification of carrier status for variants of known medical significance, characterization of risks of different diseases and identification of high-impact mutations of unknown medical significance (see Additional file 6 for advanced course syllabus). Throughout the advanced course, students were given the option of analyzing and interpreting either their own WGS data or that of an anonymous donor. Funds for conducting the sequencing were provided by the ISMMS Institute for Genomics and Multiscale Biology, and the personal genome sequence data were provided to the students free of charge.

Medical students, genetic counseling students, residents and fellows who might use the information in their practice or research and PhD students who might apply the information to their research were the target group for the course. The introductory course was advertised as a prerequisite course for the advanced human genome sequencing course in which students could have their own genomes sequenced at no cost to them with instructors blind to their decision to do so or not. Students who completed the introductory course were eligible to enroll on the advanced course and to take part in the research. The research information sheets provided to the students can be found in Additional files 7, 8 and 9. Further methodological detail can be found in Additional file 10: Supplementary Methods.

All of the 20 students who started the course were recruited to complete the questionnaires at the outset of the study, and all completed the questionnaires before and after the introductory course. Nineteen of the twenty students enrolled on the advanced course (one student was unable to attend the mandatory first class of the advanced course and so could not be enrolled). Of the 19 students who were enrolled in the advanced course, all completed the survey at the start of the advanced course. The final sample size for the present study was therefore *N* = 19. Five were genetic counseling masters students, three were medical genetics residents, three were MD/PhD students, three were PhD students, two were medical students, two were junior faculty and one was a genetics fellow. Both clinical faculty members used genetics regularly in their work and most but not all of the MD and PhD students had had some prior experience with molecular genetics or genomics in a research context. Ten of the students were male, nine were female.

This research conformed to the Declaration of Helsinki. The dean, the Research Ethics Committee (a separate body to the Institution Review Board (IRB)), which reviews issues that fall outside the remit of the IRB, and the CePORTED (Center for Patient Oriented Research, Training, Education and Development) curriculum committee at ISMMS all approved the study and/or the course. The study was submitted to the IRB, which determined that the sequencing was part of the educational experience and that the research component of the proposal (questionnaires) posed no greater than minimal risk and met criteria for exemption under Category 2 Research involving the use of educational tests or survey procedures. In addition, the course directors consulted the ISMMS general counsel and the Division of Genetics at the New York State Department of Health.

### Measures

As described in Additional file 5: Table S1 and the supplemental methods (Additional file 10), the major classes of primary variables that were assessed in the questionnaires at time points T1, T2 and T3 were: students interest in analyzing their own genome in an educational setting, including intentions, decision and discussion of decision with others; informed decision-making (this was assessed using O’Connor’s 2005 Decisional Conflict Scale and five subscales: the informed subscale, the effective decision-making (satisfaction) subscale, the support subscale, the values clarity subscale and the uncertainty subscale); attitudes towards WGS in an educational setting; attitudes towards WGS in general and knowledge regarding how to interpret DNA variants associated with disease risk in a clinical setting. We also present and describe the open-ended comments students provided at the end of the questionnaires at each of the three time points.

### Statistical analyses

We used Kolmogorov–Smirnov tests to determine that interest, decision, decisional conflict individual items, attitudes towards WGS in an educational setting and attitudes towards WGS in general were non-normally distributed. The tests showed that decisional conflict scale and subscales results were normally distributed.

The first ‘interest’ item (which assessed interest in analyzing their own genome with six response options) was recoded so that the ‘don’t know’ and ‘it depends’ response options were recoded into the midpoint. The scores were therefore: 1 = no, definitely not, 2 = no, probably not, 3 = don’t know/it depends, 4 = yes, probably, 5 = yes, definitely. This was done so that the variable could be treated as a continuous variable without discarding the ‘don’t know’ and ‘it depends’ response options. We described the variable at each time point (T1, T2 and T3) using frequencies, then conducted a non-parametric test to examine changes over time between T1 to T2, T1 to T3 and T2 to T3 because of the clearly highly skewed distribution of this variable at each time point. We used the Wilcoxon signed-rank test because the results were two non-parametric sets of scores from the same participants. We calculated the effect size *r* in Excel using the following equation in accordance with Field [21] (p. 550):

r=Z÷√N

For the ‘decision’ variable, we ran frequencies to observe and report how many respondents opted for Option 1 (analyze own genome), how many opted for Option 2 (analyze an anonymous genome) and how many did not respond to this question. We then recoded the variable so that the missing data points were recoded into Option 2 (analyze other genome). We then conducted a non-parametric test (Wilcoxon signed-rank test) as the variable was clearly skewed. Effect sizes were calculated as above. We also reported the median for this and all other items apart from the knowledge items. The 15 individual items of the decisional conflict scale (DCS) were also non-normally distributed and so were analyzed using the Wilcoxon signed-rank test as above. Effect sizes were also calculated as above.

The DCS scale and subscale were normally distributed and so changes over time on these measures were assessed using paired samples *t*-tests. The effect sizes were calculated in Excel using the following equation (Rosenthal [22]) in accordance with Field [21] (p. 332):

$$r=\sqrt{\left(t^{2}÷\left(t^{2}+\mathrm{df}\right)\right)}$$

Attitudes towards WGS in an educational setting were non-normally distributed and so were analyzed using the Wilcoxon signed-rank test as above. Effect sizes were also calculated as previously. Attitudes towards WGS in general were also non-normally distributed and so were analyzed using Wilcoxon signed-rank tests with effect sizes calculated as above. Knowledge items were dichotomous and were analyzed using Wilcoxon signed-rank tests with effect sizes calculated as above.

Effect sizes were described according to Cohen’s criteria of .3 and .5 for a medium and large effect, respectively (Field [21], p. 558). All tests were two-tailed and significance values were *P* < 0.05. All statistical analyses were conducted using IBM SPSS version 19 (Chicago, IL), with the exception of the effect sizes which were calculated using Excel 2010.

## Results

### Aim 1: To assess key whole genome sequencing decision-related attitudes and knowledge at the start of an introductory genomics course (T1)

#### Interest and decision

**Figure 1:**
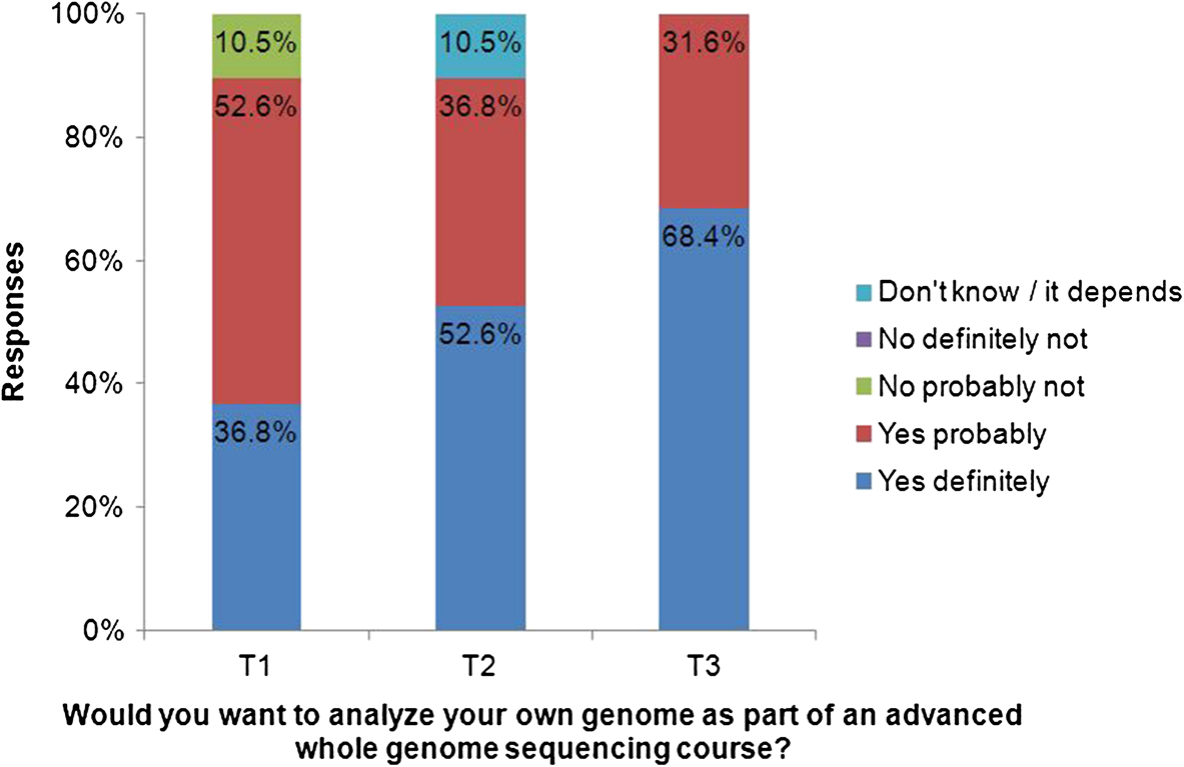
**Trend relating to desire to analyze one’s personal genome in an advanced genome sequencing course.** This was observed at three time points before an introductory genomics course (T1), after the introductory genomics course (T2) and before the subsequent advanced genomics course (T3) taken by 19 students at the Icahn School of Medicine at Mount Sinai during the 2012 summer and fall semesters. Desire to analyze their own genome did not change between T1 and T2 (Wilcoxon signed-rank test *P* = 0.16), but significantly increased between T1 and T3 (Wilcoxon signed-rank test *P* = 0.014), and between T2 and T3 (Wilcoxon signed-rank test *P* = 0.025).

At baseline, 17 of the 19 students selected ‘Option 1: I would like to analyze my own genome as part of an advanced WGS course’, while the remaining two students selected ‘Option 2: I would not like to analyze my own genome as part of an advanced WGS course, and would rather analyze an anonymous donated genome’ (see Table 1). Similarly, 17 of the 19 students said that they would ‘probably’ or ‘definitely’ want to analyze their own genomes as part of the advanced WGS course when presented with six response options ranging from ‘no, definitely not’ to ‘yes, definitely.’ However, it is worth noting that the median response was ‘yes, probably’ and that only seven students selected ‘yes, definitely’ at this time point (see time point T1 in Figure 1).

**Table 1:** Students’ interest in analyzing their own genomes in an educational setting

***N*** **= 19**	**Response options**	**T1**	**T2**	**T3**	**Significance**^**c**^		
**Interest/decision**		***N *** **(%)**	***N (*%)**	***N (*%)**	**T1 to T2**^**a**^	**T1 to T3**^**a**^	**T2 to T3**^**a**^
Would you want to analyze your own genome as part of an advanced WGS course?	No, definitely not	0 (0%)	0 (0%)	0 (0%)	*z* = −1.41, *P* = 0.16, *r* = −0.32	***z*** **= −2.46, *P*** **= 0.014, *r*** **= −0.56**	***z*** **= −2.24, *P*** **= 0.025, *r*** **= −0.51**
	No, probably not	2 (10.5%)	0 (0%)	0 (0%)			
	Don’t know/It depends	0 (0%)	2 (10.5%)	0 (0%)			
	Yes, probably	10 (52.6%)	7 (36.8%)	6 (31.6%)			
	Yes, definitely	7 (36.8%)	10 (52.6%)	13 (68.4%)			
	Median	Yes, probably	Yes, definitely	Yes, definitely			
At this point, which of the following options would you prefer? Please check one.	Option 1: I would like to analyze my own genome as part of an advanced WGS course.	17 (89.5%)	18 (94.7%)	18 (94.7%)	*z* = −1.41, *P* = 0.16, *r* = −0.32	*z* = −0.58, *P* = 0.56, *r* = −0.13	*z* = −1.00, *P* = 0.32, *r* = −0.23
	Option 2: I would not like to analyze my own genome as part of an advanced WGS course, and would rather analyze an anonymous donated genome.	2 (10.5%)	0 (0%)	0 (0%)			
	[Participant did not check either option]	0 (0%)	1 (5.3%)	1 (5.3%)			
**Decisional conflict: individual items (agree/strongly agree)**		**N (%)**	**N (%)**	**N (%)**	**T1 to T2**^**a**^	**T1 to T3**^**a**^	**T2 to T3**^**a**^
I know which options are available to me.		17 (89.5%)	19 (100%)	18 (94.7%)	*z* = −1.90, *P* = 0.058, *r* = −0.44	***z*** **= −2.45, *P*** **= 0.014, *r*** **= −0.56**	*z* = 0.00, *P* = 0.99, *r* = 0.00
Median		Agree	Strongly agree	Strongly agree			
I know the benefits of each option.		13 (68.4%)	18 (94.7%)	18 (94.7%)	***z*** **= −2.57, *P*** **= 0.010, *r*** **= −0.59**	***z*** **= −3.00, *P*** **= 0.003, *r*** **= −0.69**	***z*** **= −2.00, *P*** **= 0.046, *r*** **= −0.46**
Median		Agree	Agree	Strongly agree			
I know the risks of each option.		9 (47.4%)	17 (89.5%)	18 (94.7%)	***z*** **= −2.86, *P*** **= 0.004, *r*** **= −0.66**	***z*** **= −3.41, *P*** **= 0.001, *r*** **= −0.78**	*z* = −1.34, *P* = 0.18, *r* = 0.31
Median		Neither	Agree	Agree			
I am clear about which benefits matter most to me.		10 (55.6%)	16 (84.2%)	18 (94.7%)	***z*** **= −2.51, *P*** **= 0.012, *r*** **= −0.58**	***z*** **= −2.80, *P*** **= 0.005, *r*** **= −0.64**	*z* = −0.38, *P* = 0.71, *r* = −0.08
Median		Agree	Agree	Agree			
I am clear about which risks matter most.		9 (47.4%)	16 (84.2%)	17 (89.5%)	***z*** **= −2.41, *P*** **= 0.016, *r*** **= −0.55**	***z*** **= −2.38, *P*** **= 0.017, *r*** **= −0.55**	*z* = −0.07, *P* = 0.94, *r* = −0.02
Median		Neither	Agree	Agree			
I am clear about which is more important to me (the benefits or the risks).		11 (57.9%)	16 (84.2%)	17 (89.5%)	*z* = −1.51, *P* = 0.13, *r* = −0.35	***z*** **= −2.23, *P*** **= 0.026, *r*** **= −0.53**	*z* = −1.67, *P* = 0.096, *r* = 0.38
Median		Agree	Agree	Agree			
I have enough support from others to make a choice.		10 (52.6%)	18 (94.7%)	15 (78.9%)	***z*** **= −2.88, *P*** **= 0.004, *r*** **= −0.66**	***z*** **= −2.12, *P*** **= 0.034, *r*** **= −0.49**	*z* = −0.92, *P* = 0.36, *r* = −0.21
Median		Agree	Agree	Agree			
I am choosing without pressure from others.		18 (94.7%)	17 (89.5%)	17 (89.5%)	*z* = −0.63, *P* = 0.53, *r* = −0.14	*z* = −0.82, *P* = 0.41, *r* = 2.25	*z* = 0.00, *P* = 0.99, *r* = 0.00
Median		Agree	Strongly agree	Strongly agree			
I have enough advice to make a choice.		10 (52.6%)	14 (73.7%)	17 (89.5%)	***z*** **= −2.44, *P*** **= 0.015, *r*** **= −0.56**	***z*** **= −3.09, *P*** **= 0.002, *r*** **= −0.71**	*z* = −1.73, *P* = 0.083, *r* = −0.40
Median		Agree	Agree	Agree			
I feel sure about what to choose.		11 (57.9%)	13 (68.4%)	15 (78.9%)	*z* = −1.21, *P* = 0.23, *r* = −0.28	*z* = −1.96, *P* = 0.051, *r* = 0.45	*z* = −1.16, *P* = 0.25, *r* = −0.27
Median		Agree	Agree	Agree			
This decision is easy for me to make.		8 (42.1%)	12 (63.2%)	13 (68.4%)	*z* = −1.73, *P* = 0.083, *r* = −0.40	***z*** **= −2.51, *P*** **= 0.012, *r*** **= 4.36**	*z* = −1.40, *P* = 0.16, *r* = −0.32
Median		Neither	Agree	Agree			
I feel I have made an informed choice.		9 (47.4%)	16 (84.2%)	19 (100%)	***z*** **= −2.88, *P*** **= 0.004, *r*** **= −0.66**	***z*** **= −2.97, *P*** **= 0.003, *r*** **= −0.68**	*z* = −1.13, *P* = 0.26, *r* = −0.26
Median		Neither	Agree	Agree			
My decision shows what is important to me.		12 (63.2%)	18 (94.7%)	15 (78.9%)	***z*** **= −2.39, *P*** **= 0.017, *r*** **= −0.55**	***z*** **= −2.33, *P*** **= 0.020, *r*** **= 4.36**	*z* = −0.38, *P* = 0.71, *r* = 0.08
Median		Agree	Agree	Agree			
I expect to stick with my decision.		14 (73.7%)	15 (78.9%)	17 (89.5%)	*z* = −0.92, *P* = 0.36, *r* = −0.21	***z*** **= −2.17, *P*** **= 0.030, *r*** **= 0.50**	*z* = −1.13, *P* = 0.26, *r* = −0.26
Median		Agree	Agree	Strongly agree			
I am satisfied with my decision.		12 (63.2%)	15 (78.9%)	17 (89.5%)	***z*** **= −2.65, *P*** **= 0.008, *r*** **= −0.61**	***z*** **= −2.71, *P*** **= 0.007, *r*** **= 0.62**	*z* = −1.00, *P* = 0.32, *r* = −0.23
Median		Agree	Agree	Agree			
**Decisional conflict: overall scale and subscales**		**Mean (SD), range**	**Mean (SD), range**	**Mean (SD), range**	**T1 to T2**^**b**^	**T1 to T3**^**b**^	**T2 to T3**^**b**^
Overall decisional conflict scale		33.88 (18.18), 0.00 to 70.31	19.82 (14.24), 0.00 to 46.88	16.61 (14.55), 0.00 to 43.75	***t*(18) = 3.99, *P*** **= 0.001, *r*** **= 0.69**	***t*(18) = 4.83, *P*** **< 0.001, *r*** **= 0.75**	*t*(18) = 1.72, *P* = 0.10, *r* = 0.38
Subscales:							
Informed subscale		31.14 (21.31), 0.00 to 75.00	15.35 (12.19), 0.00 to 41.67	12.28 (13.99), 0.00 to 50.00	***t*(18) = 3.66, *P*** **= 0.002, *r*** **= 0.65**	***t*(18) = 4.88, *P*** **< 0.001, *r*** **= 0.75**	*t*(18) = 1.79, *P* = 0.090, *r* = 0.39
Effective decision-making (satisfaction) subscale		31.58 (19.82), 0.00 to 68.75	19.08 (15.23), 0.00 to 50.00	16.12 (15.63), 0.00 to 43.75	***t*(18) = 3.78, *P*** **= 0.001, *r*** **= 0.67**	***t*(18) = 4.00, *P*** **= 0.001, *r*** **= 0.69**	*t*(18) = 1.18, *P* = 0.25, *r* = 0.27
Support subscale		31.13 (17.97), 0.00 to 66.67	16.67 (14.43), 0.00 to 50.00	15.79 (17.10), 0.00 to 50.00	***t*(18) = 3.43, *P*** **= 0.003, *r*** **= 0.63**	***t*(18) = 3.36, *P*** **= 0.003, *r*** **= 0.62**	*t*(18) = 0.36, *P* = 0.73, *r* = 0.08
Values clarity subscale		35.09 (24.78), 0.00 to 75.00	20.18 (15.79), 0.00 to 50.00	17.11 (15.08), 0.00 to 50.00	***t*(18) = 2.79, *P*** **= 0.012, *r*** **= 0.55**	***t*(18) = 3.23, *P*** **= 0.005, *r*** **= 0.61**	*t*(18) = 0.94, *P* = 0.36, *r* = 0.22
Uncertainty subscale		40.79 (28.21), 0.00 to 75.00	32.24 (29.85), 0.00 to 100.00	25.00 (23.57), 0.00 to 75.00	*t*(18) = 1.95, *P* = 0.067, *r* = 0.42	***t*(18) = 3.13, *P*** **= 0.006, *r*** **= 0.59**	*t*(18) = 1.57, *P* = 0.13, *r* = 0.35

^a^Differences assessed using Wilcoxon signed-rank test.

^b^Differences assessed using paired samples *t*-tests.

^c^Bold text in the Significance column highlights results that were significant at the *P* < 0.05 level.

#### Decisional conflict (scale and subscales)

**Figure 2:**
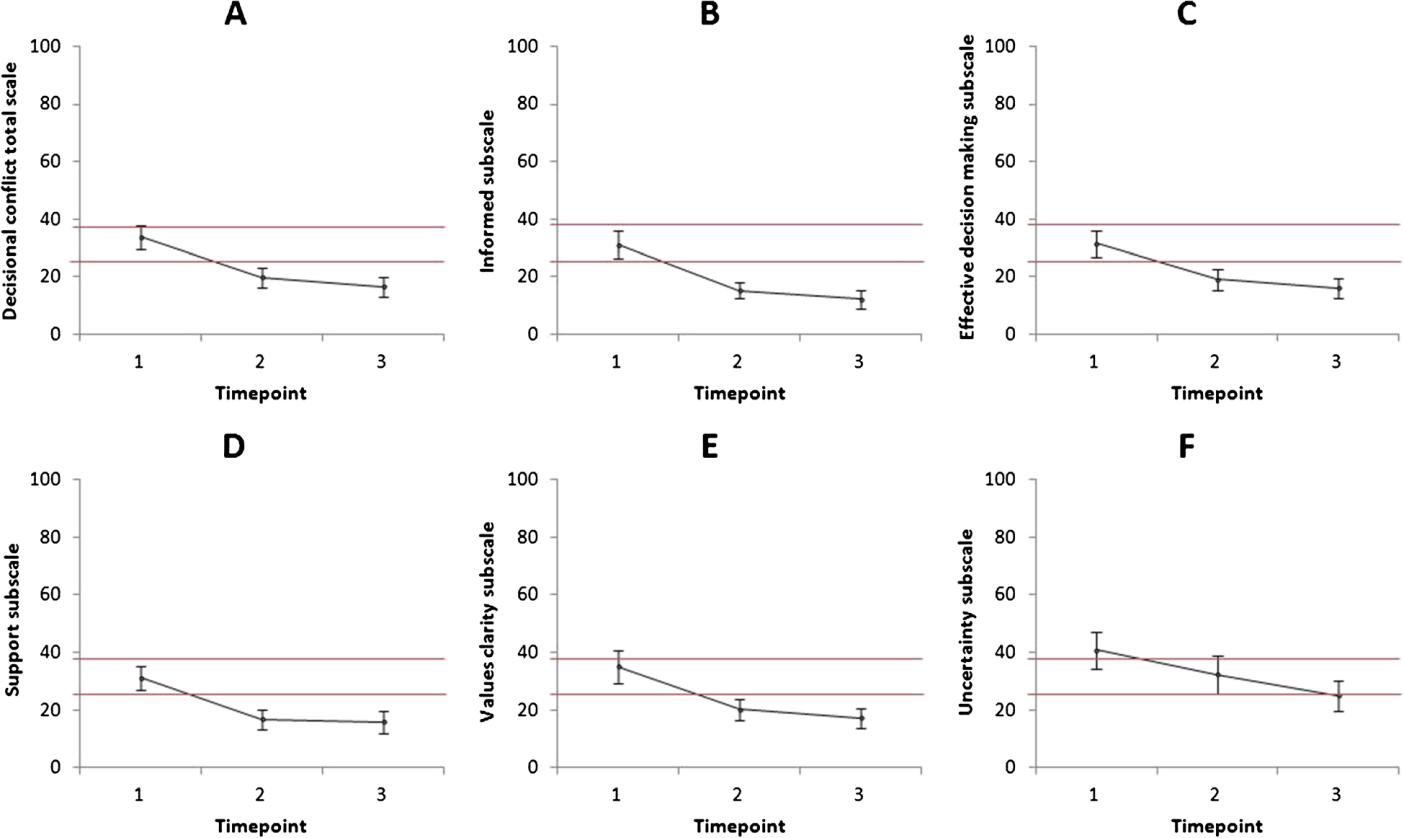
**Trends relating to decisional conflict overall scale and five subscales about using one’s personal genome in an advanced genome sequencing course.** These were observed as part of a two-part genomics course taken by 19 students at the Icahn School of Medicine at Mount Sinai in 2012. All *P* values were calculated using paired samples *t*-tests. Lower scores indicate lower levels of decisional conflict. Scores are means with standard error bars displayed. **(A)** Decisional conflict about whether or not to get one’s own genome sequenced decreased over time between T1 and T2 (*P* < 0.001) and T1 and T3 (*P* < 0.001). The difference was not significant between T2 and T3 (*P* = 0.10). **(B)** Scores on the informed subscale decreased between T1 and T2 (*P* = 0.002) and between T1 and T3 (*P* < 0.001) indicating students felt more informed at T2 and T3 than they did at T1. **(C)** Scores on the effective decision-making subscale decreased between T1 and T2 (*P* = 0.001) and between T1 and T3 (*P* = 0.001), indicating students felt more satisfied with their decisions at T2 and T3 than they did at T1. **(D)** Scores on the support subscale decreased between T1 and T2 (*P* = 0.003) and between T1 and T3 (*P* = 0.003), indicating students felt more supported at T2 and T3 than they did at T1. **(E)** Scores on the values clarity subscale decreased between T1 and T2 (*P* = 0.012) and between T1 and T3 (*P* = 0.005), indicating students felt greater clarity about their values at T2 and T3 than they did at T1. **(F)** Scores on the uncertainty subscale decreased between T1 and T3 (*P* = 0.006), indicating students felt less uncertainty about their decisions at T3 than they did at T1.

At baseline, the mean overall decisional conflict scale score was 33.9 ± 18.2 (range 0.0 to 70.3). This was lower than the cut-off of 37.5 (scores exceeding 37.5 are associated with decision delay or feeling unsure about implementation), but exceeded the cut-off of 25 (scores lower than 25 are associated with implementing decisions), suggesting that the students were experiencing some conflict around their decision regarding whether or not to have their genomes sequenced as part of the advanced WGS course. The mean scores for all five decisional conflict subscales similarly exceeded 25 at baseline (see Table 1 and Time point 1 in Figure 2).

#### Decisional conflict (individual items)

**Figure 3:**
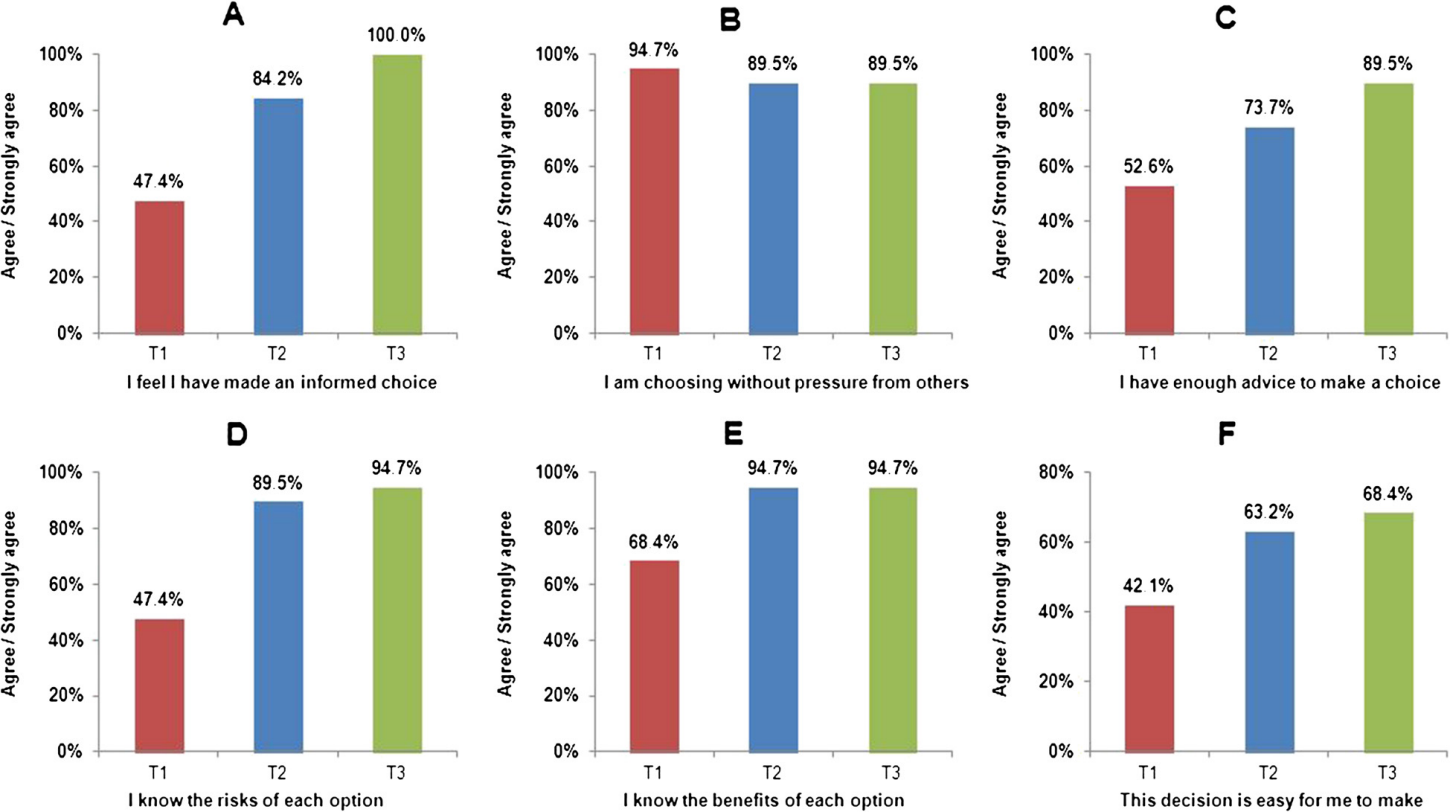
**Trends relating to decisional conflict individual items. (A)** I feel I have made an informed choice. **(B)** I am choosing without pressure from others. **(C)** I have enough advice to make a choice. **(D)** I know the risks of each option. **(E)** I know the benefits of each option. **(F)** This decision is easy for me to make.

As Figure 3A shows, only 47% of the students stated that they were making an ‘informed choice’ at baseline. Relatedly, only 47% felt they knew the risks of each option, only 42% felt that the decision was easy for them to make and only 53% felt they had enough advice to make a choice. Further, 68% stated that they knew the benefits of each option. Of the students, 95% (18 out of the 19) stated that they were ‘choosing without pressure from others’ (see time point T1 in Figures 3B–F; also see Table 1).

#### Attitudes towards whole genome sequencing in an educational setting

At baseline, 16 of the 19 students believed that analyzing their own genomes as part of the advanced WGS course ‘would be useful’, and all 19 of the students agreed or strongly agreed that an important reason in favor of using their own genome was to ‘satisfy general curiosity’. The students endorsed a number of additional reasons for wanting to analyze their own genomes, including determining if specific diseases run in their family or are DNA based, helping understand principles of human genetics and enhancing their understanding of what a patient may learn or experience by having their genomes sequenced (see Additional file 5: Table S2). Sixteen students agreed or strongly agreed that they would see this as an opportunity to get a service that they would not ordinarily get if they had to pay full price. Only two students stated that they would be concerned that their professors ‘would know who took up the offer of testing and who did not’. Some additional concerns were endorsed, with ten students expressing concern about privacy or risks to privacy, and 15 students expressing concern that they might get some results that would be disturbing. See Additional file 5: Table S2 for the complete results regarding attitudes towards WGS in an educational setting at baseline.

#### Attitudes towards whole genome sequencing in general

Students had wide-ranging general views regarding WGS (see Additional file 5: Table S3). For example, a majority of the students felt that results of WGS would influence health-care decisions, but none of the students agreed that ‘most people can accurately interpret WGS results’. Fewer than half agreed that WGS results would be useful to a physician. In line with the decisional conflict items, only 11 of the 19 students reported that they understood the risks and benefits of getting personal WGS done (see Additional file 5: Table S3).

#### Knowledge

Fewer than half of the students correctly interpreted the information provided in the clinical scenarios that were used to assess knowledge, indicating low levels of knowledge at baseline (Additional file 5: Table S4). Scores were similarly low on the other knowledge measures assessing what factors influenced their understanding and how they would counsel a patient (Additional file 5: Table S4).

### Aim 2: To assess changes over time in key decision-related attitudes and knowledge between the start and end of an introductory genomics course (T1 to T2)

#### Interest and decision

At the end of the introductory course, ten students stated that they would ‘definitely’ like to analyze their own genomes, compared to the seven students who stated this at baseline. This was not a statistically significant change, although it is worth noting that this was nonetheless a medium effect size (*z* = −1.41, *P* = 0.16, *r* = 0.32). See Table 1 for the full interest/decision results.

#### Decisional conflict (scale and subscales)

There was a significant decrease between baseline and the end of the introductory course (T1 to T2) in decisional conflict overall, and the effect size was large (*t*(20) = 3.66, *P* = 0.002, *r* = 0.69). Similarly, there were significant changes between T1 and T2 on four of the five decisional conflict subscales, all with large effect sizes with *r* > 0.5, indicating that the students felt significantly more informed, more satisfied and more supported and had greater clarity about their values after compared to before the introductory course (see Figure 2). There was also a medium effect size for the uncertainty subscale, although this did not reach statistical significance (see Table 1).

#### Decisional conflict (individual items)

Also as shown in Table 1, examination of the individual decisional conflict items revealed that there were several significant shifts between T1 and T2. For example, the proportion of students who stated they were making ‘an informed choice’ increased from 47% at the start of the introductory course to 84% at the end of the introductory course (*z* = −2.88, *P* = 0.004, *r* = −0.66). Similarly, the proportion who stated that they knew ‘the risks’ increased from 47% to 90% (*z* = −2.86, *P* = 0.004, *r* = −0.66) (see also Figure 3).

#### Attitudes towards whole genome sequencing in an educational setting

The students’ attitudes towards WGS in an educational setting mainly did not change significantly during the introductory course. However, there were a few notable exceptions. By the end of the introductory course, fewer students were concerned about privacy (*z* = −2.39, *P* = 0.017, *r* = 0.55) and fewer were concerned that people would find out genetic or health information about them (*z* = −2.49, *P* = 0.013, *r* = 0.57). Also, a greater number of students believed that their own results would help them understand genetic concepts better than someone else’s results (*z* = −2.12, *P* = 0.034, *r* = 0.49). (See Additional file 5: Table S2).

#### Attitudes towards whole genome sequencing in general

As shown in (Additional file 5: Table S3), the students’ attitudes towards WGS in general also did not change significantly during the introductory course, with the exception that significantly more students felt that they understood the risks and benefits of personal genome sequencing by the end of the course (*z* = −2.71, *P* = 0.007, *r* = 0.62). The observed changes were largely maintained over time, in that the differences remained significant in the analyses comparing the start of the introductory course with the start of the advanced course (T1 to T3; see Additional file 5: Table S3).

### Knowledge

Knowledge regarding interpretation of clinically presented genomic test results did not change from before to after the introductory course (see Additional file 5: Table S4).

### Aim 3: To assess changes over time in key decision-related attitudes and knowledge between the end of an introductory course and start of an advanced genomics course (T2 to T3)

As shown in Table 1, Additional file 5: Tables S2, S3 and S4, and Figures 2 and 3, there were few changes over time between the end of the introductory and start of the advanced genomics course (T2 to T3). When asked with whom they had discussed their decision about whether to get their genome sequenced, 12 students stated they had discussed the decision with their friends, ten with their spouse or significant other, eight with their mother and two with a genetic counselor (see Table 2). All of the students stated that they ‘probably’ or ‘definitely’ intended to have blood drawn for genome sequencing and to analyze their genome sequence as part of the advanced course. Seven students stated that they ‘probably’ or ‘definitely’ were going to talk to a genetic counselor about their decision. Based on the course records, all 19 of the students subsequently chose to have blood drawn and analyze their own genome data as part of the advanced course, and only two students talked to a genetic counselor prior to making this decision (see Table 2).

**Table 2:** Discussion of the decision with others and intentions at T3, and actual decisions made subsequently

***N*** **= 19**		***N *** **(%)**
**Discussion of decision with others, self-reported at T3**		
Have you discussed whether or not to get your genome sequenced as part of this course with anyone?	Yes	17 (89.5%)
	No	2 (10.5%)
Who have you talked to about whether or not to get your genome sequenced as part of this course?	Genetic counselor	2 (10.5%)
	Other health professional	2 (10.5%)
	Mother	8 (42.1%)
	Father	7 (36.8%)
	Brother/sister	3 (15.8%)
	Other family member	3 (15.8%)
	Friend(s)	12 (63.2%)
	Spouse/significant other	10 (52.6%)
	One or more of the course directors	1 (5.3%)
	Other (if other, please specify)^a^	1 (5.3%)
**Intentions, self-reported at T3**		
I intend to have the blood draw for WGS as part of this course	No, definitely not	0 (0%)
	No, probably not	0 (0%)
	Don’t know/it depends	0 (0%)
	Yes, probably	6 (31.6%)
	Yes, definitely	13 (68.4%)
	Median	Yes, definitely
I intend to analyze my own genome as part of this WGS course	No, definitely not	0 (0%)
	No, probably not	0 (0%)
	Don’t know/it depends	0 (0%)
	Yes, probably	6 (31.6%)
	Yes, definitely	13 (68.4%)
	Median	Yes, definitely
I intend to seek genetic counseling before making my decision about whether to obtain my own personal genome sequence data	No, definitely not	5 (26.3%)
	No, probably not	7 (36.8%)
	Don’t know/it depends	0 (0%)
	Yes, probably	2 (10.5%)
	Yes, definitely	5 (26.3%)
	Median	No, probably not
**Observed actual behaviors, after T3**		
Did the student actually have their blood drawn and analyze their own genome as part of the WGS course?		
Yes		19 (100%)
Did the student see the genetic counselor provided within the medical school prior to receiving their WGS data?		
Yes		2 (10.5%)
Did the student see the genetic counselor provided outside of the medical school prior to receiving their WGS data?		
Yes		0 (0%)

^a^The ‘other’ response here was: ‘Just mentioned it not looking for an answer because there is actually no question here. I will meet with a genetic counselor though just to learn about the experience’.

#### Students’ responses to open-ended comments section

Here we briefly describe the students’ responses to the open-ended comments sections at the end of each of the three questionnaires, focusing particularly on responses related to the students’ views on personal WGS.

At the beginning of the introductory course (T1), students’ responses to the open-ended comments section of the questionnaire reflected two main themes: concerns about personal WGS (expressed by four students) and questions about personal WGS (expressed by three students). The concerns largely mapped onto those listed in the quantitative part of the questionnaire, and included concerns about treatments or cures for many genetic diseases being unavailable, privacy and who would have access to the WGS results and implications for life insurance. One student particularly expressed fear of detecting predisposition to disease, while another thought that analyzing their own genome might not be more educational and could be disturbing. Specific questions raised included whether genetic counselling would be available before or after personal WGS, whether course directors would know the results of personal WGS and whether an information letter would be provided for family members.

At the end of the introductory course (T2), some students again raised concerns about personal WGS (three students in total), including concerns about privacy and ownership of data. One student in particular stated that the decision to sequence their own genome or not remained very difficult for them due to many variants being difficult to interpret, and because they remained fearful of finding variants that carried significant disease risk. One student stated that they thought a ‘mock’ genome would be just as useful to their learning experience. Two students expressed excitement about getting their own genomes sequenced. Whereas a number of questions had been raised about personal WGS at the start of the course, only one student raised questions at the end of the course, and these related to privacy, ownership of genomic information and publication or access to the genomic information by third parties. Several students expressed positive views of the introductory course.

At the start of the advanced course (T3), no concerns were expressed or questions raised in this section of the questionnaire. See Additional file 5: Table S5 for all of the students’ responses to the open-ended questionnaire sections.

## Discussion

The results of our study suggest that students enrolled in an introductory WGS course were interested in analyzing their own genomes. This interest did not appear to be affected by learning more about the risks and limitations of WGS and the course successfully reduced the degree of uncertainty students felt about their decision whether or not to sequence their own genomes. As a result of the introductory genomics course, it appears that the majority of the students made more informed decisions about sequencing their own genomes. On average they felt more informed, more clear about their personal values regarding benefits and risks, more supported in their decision-making and more satisfied with their decision after, compared to before, the course. This suggests that the introductory course improved the extent to which students were able to make informed decisions about getting their genomes sequenced and they were decisions that they were comfortable with.

Although the course made students feel that they were making a more informed decision as measured by the variables in the Decisional Conflict Scale, when we attempted to measure objectively whether knowledge about genomics concepts increased from pre- to post-course we found no significant changes over time. This could be because we utilized a set of three scenario-based questions that were developed by the group at Stanford [23] and which therefore focused more on understanding and interpretation of consumer genomics test results, rather than being developed specifically to tap acquired knowledge about the topics covered in our WGS course. Measuring knowledge about genomics is notoriously difficult, and there were few alternative existing measures at the time that we could have used. Although one new measure has now been developed for use with general populations [24], it is not clear that this measure would be appropriate for use with students learning about WGS at a higher level. Further validation of the measure by Kaphingst and colleagues in different populations is needed, and new measures of knowledge about WGS that can be used with students of genomics need to be developed. It may also be argued that objectively assessed knowledge about genomics and WGS is not an essential prerequisite for informed decision-making about personal genome sequencing, and that feeling informed about the risks and benefits is more important in this context.

In all areas of life, what people say they will do does not always map on to what they actually subsequently do. This is as true in personal genomics and genetic testing as anywhere else. When surveyed, a majority of individuals say that they are interested and would obtain personal genetic or genomic information about themselves if given the opportunity [25-30], yet invariably, fewer individuals actually go on to have genomic or genetic testing when it really is presented to them as an option [26,27,29]. This diminished interest in the face of making an actual decision may be attributable to the individual learning more about the risks, limitations and uncertainties of the technology, and thinking through more carefully what the actual psychosocial, financial and other potentially adverse outcomes might be for them personally, as well as practical barriers. Often, genetic counseling is a key facilitator of this more deliberative process. In an educational context, students who opt to take courses in which they are given the option of obtaining personal genomic information free of charge are likely to be a highly self-selected sample with considerable interest in getting their genomes sequenced at baseline. However, previous evidence from other contexts suggests that even individuals who are initially interested in receiving personal genetic or genomic information often lose interest when they learn more about the risks and limitations or think through the implications for them personally more carefully. In a survey of students attending a genomics course (in which personal genomic data was not offered) at Stanford University, the students expressed considerable enthusiasm for personal genomics, but their enthusiasm decreased over time as they learnt more during the course [23]. This emphasizes the likely importance of providing students with sufficient information, time and support to make considered and informed personal decisions about whether to get their genomes sequenced as part of their educational process.

In the present study, our findings do indeed suggest that providing information, time and support may be important in helping students make informed decisions about receiving their personal genome sequence data as part of their education. However, it is also important to note that the students in our study were offered their personal genome sequence data free of charge. This differs from the Stanford study in which students paid towards their 23andMe genetic test results [12,13]. The fact that the students on our course were offered something that is not widely available – their entire personal genome sequence – at no financial cost to them, could have had a considerable impact on how they weighed the benefits vs. risks of receiving, and on their ultimate decisions to receive, their personal genomes. This is supported by our finding that most students agreed with the statement that getting WGS in an educational setting would be ‘an opportunity that I would not ordinarily get if I had to pay full price’. The fact that the students in our study were able to obtain and keep their personal genome sequence data at no financial cost to them, could clearly have been a significant incentive to them to participate and have their genomes sequenced, perhaps trumping even significant concerns or worries about risks that they might have been feeling.

The primary limitations of our study included that this was a single site study with a small sample size, and that due to the preliminary nature of the study we did not correct for multiple comparisons in the statistical analyses. The small number of individuals included in the study could clearly leave us underpowered to detect differences that we would have otherwise detected had we had a larger sample size. However, it was arguably appropriate to conduct this first-of-a-kind study on a small scale that could be more controlled (for example, by being more likely to be alerted to problems than if there were a larger number of students involved), before considering scaling up with a larger number of students. Perhaps more important is the self-selected nature of this group of students. Some responded to an email advertising the course, and some were informed about the course because they were either within or connected to the Department of Genetics and Genomic Sciences. These students may very likely have had greater enthusiasm for personal genome sequencing, both by nature of being self-selected on to the course, and because of the novelty of the course at the time it was offered, than other students in other contexts are likely to have in the future. The results are therefore not generalizable to other students, and would need to be replicated in samples of students with differing characteristics, for example, at different sites and from different disciplinary backgrounds, before general conclusions could be drawn.

We recognize that there could have been some under-reporting of some variables, for example, whether the students felt pressured by others or felt unable to be critical of the course out of concern for how this might affect their assessment by the course directors or their interactions with their peers. In addition, because there was no control group, we cannot directly infer causality. The changes over time could conceivably have occurred due to factors outside the context of the course. However, due to the study design, we were able to compare changes over time before and after the introductory course to changes over time in which students were not attending genomics classes: the results suggested that it was the content of the course that successfully increased the extent to which the students felt they were making informed decisions about whether or not to analyze their own genomes, rather than simply the passing of time. Conducting this study or one like it with a control group would certainly further reduce the potential for confounding factors to play a role, although this would be challenging within an educational setting.

Finally, it is not clear whether 26 hours of teaching prior to decision-making is necessary, or whether fewer hours would lead to similar shifts in informed decision-making. However, despite the limitations and continuing concerns regarding ethical and legal issues, our study provides a framework for others to carry out studies on personal genome use in medical genomic courses, and suggests that significant efforts to increase informed decision-making among students are worthwhile.

## Conclusions

In the present study, we examined informed decision-making and related outcomes among students who were considering having their genomes sequenced as part of a novel WGS course. There are concerns, both practical and ethical, about enabling students to analyze their own personal genomes for educational purposes if they so choose. The results of our study provide empirical evidence to inform the debate on this issue and suggest that the majority of students on this WGS course were able to make independent, informed choices. Most of these students did not feel pressured into using their own genomes, and were comfortable with their decisions. While recognizing the limitations of this small study, we conclude that, given appropriate levels of support, information and time, the majority of students are likely to make informed choices about having their personal genomes sequenced.

## Abbreviations

DCS: Decisional Conflict Scale; FDA: Food and Drug Administration; IRB: Institution Review Board; ISMMS: Icahn School of Medicine at Mount Sinai; SNP: single nucleotide polymorphism; WGS: whole genome sequencing.

## Competing interests

The authors declare that they have no competing interests.

## Authors’ contributions

SCS conceived the study, participated in the design of the study, performed the statistical analysis and drafted the manuscript. ML participated in the design of the study and the coordination of the questionnaire administration and sequencing. AK participated in the design of the study and its coordination. AB participated in the design of the study and the sequencing. GD participated in the design of the study. MM participated in the design of the study and the sequencing. HS participated in the sequencing. MW participated in the design of the study. RZ participated in the design of the study and in the coordination of the genetic counseling offered to the students. MZ participated in the questionnaire design and data collection. ES conceived the study, participated in its design and coordination and helped to draft the manuscript. AK, AB, GD, ML and RZ co-developed and co-directed the courses. MDL was the lead instructor and course organizer. All authors read and approved the final manuscript.

## Supplementary Material

Additional file 1: Figure S1 Overarching conceptual framework for informed decision-making and impact of personal WGS in an educational setting. Click here for file

Additional file 2: Figure S2 Flow chart of entire study. Click here for file

Additional file 3 Questionnaire administered before and after the introductory course (T1 and T2). Click here for file

Additional file 4 Questionnaire administered at the start of the advanced course (T3). Click here for file

Additional file 5: Table S1 Quantitative measures assessed in entire study. **Table S2:** Students’ interest in analyzing their own genomes in the educational setting and uncertainty (conflict) around that decision at T1, T2 and T3. **Table S3:** Students’ attitudes towards personal whole genome sequencing in general. **Table S4:** Students’ knowledge about personal genomic results based on three clinical scenarios. **Table S5:** Students’ responses to the open-ended questions comments sections of the questionnaires. Click here for file

Additional file 6 Syllabus. Click here for file

Additional file 7 Participant information sheet for the research component including the T1 and T2 questionnaires. Click here for file

Additional file 8 Participant information sheet for the research component including the T3 questionnaire. Click here for file

Additional file 9 Information sheet for the personal whole genome sequencing component of the ‘Practical analysis of your personal genome’ course. Click here for file

Additional file 10 Supplementary methods. Click here for file
