# Supplementary material for: Informed decision-making among students analyzing their personal genomes on a whole genome sequencing course: a longitudinal cohort study
Source: Genome Med. 2013 Dec 30;5(12):113. doi: 10.1186/gm518 (PMC3971344; doi:10.1186/gm518)
Supplement: Additional file 1: Figure S1 — Overarching conceptual framework for informed decision-making and impact of personal WGS in an educational setting. [file gm518-S1.pptx]

## Slide 1
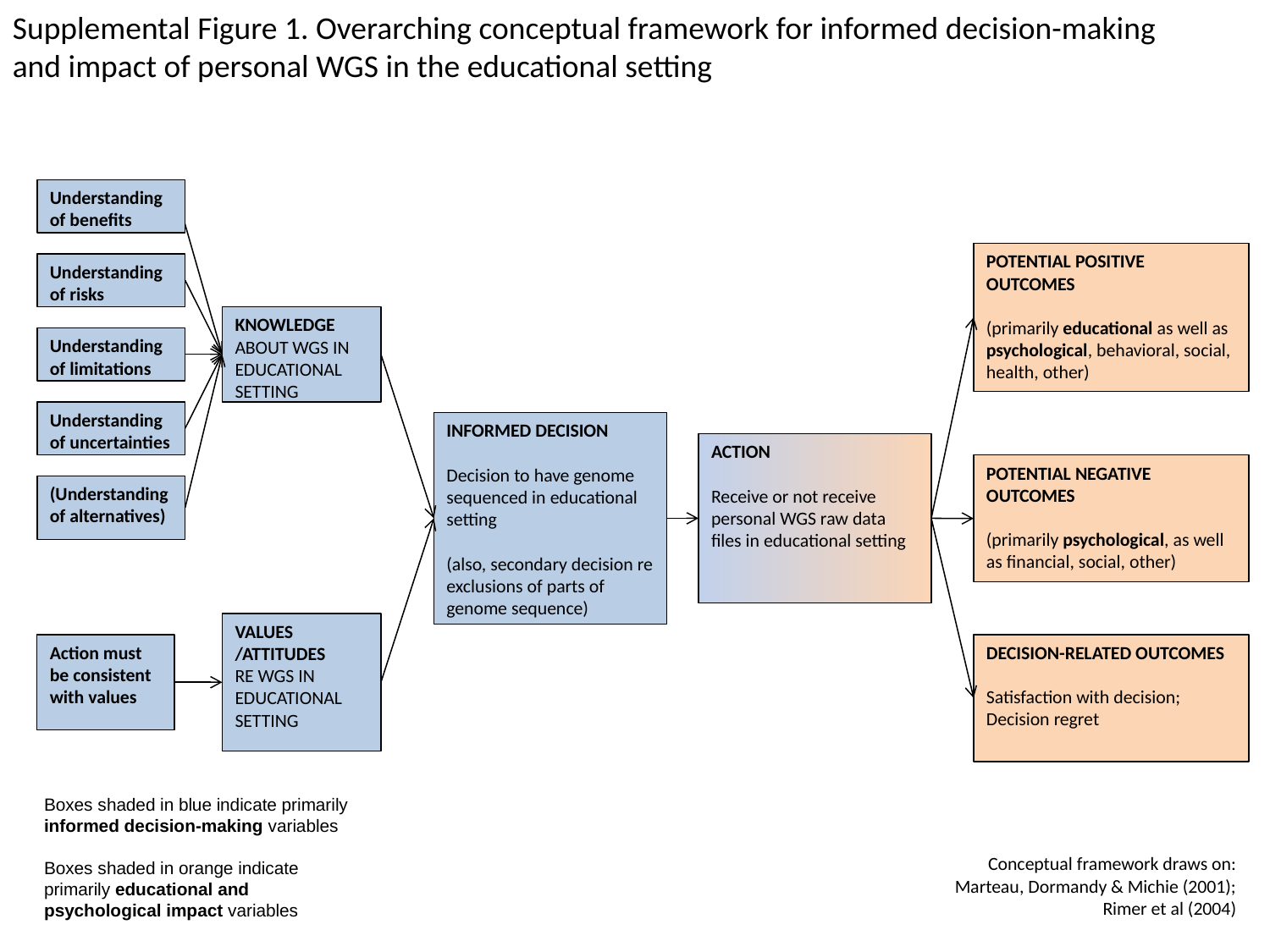

Supplemental Figure 1. Overarching conceptual framework for informed decision-making and impact of personal WGS in the educational setting
Understanding of benefits
POTENTIAL POSITIVE OUTCOMES
(primarily educational as well as psychological, behavioral, social, health, other)
Understanding of risks
KNOWLEDGE ABOUT WGS IN EDUCATIONAL SETTING
Understanding of limitations
Understanding of uncertainties
INFORMED DECISION
Decision to have genome sequenced in educational setting
(also, secondary decision re exclusions of parts of genome sequence)
ACTION
Receive or not receive personal WGS raw data files in educational setting
POTENTIAL NEGATIVE OUTCOMES
(primarily psychological, as well as financial, social, other)
(Understanding of alternatives)
VALUES
/ATTITUDES
RE WGS IN EDUCATIONAL SETTING
Action must be consistent with values
DECISION-RELATED OUTCOMES
Satisfaction with decision; Decision regret
Boxes shaded in blue indicate primarily informed decision-making variables
Boxes shaded in orange indicate primarily educational and psychological impact variables
Conceptual framework draws on: Marteau, Dormandy & Michie (2001); Rimer et al (2004)
