# Supplementary material for: Informed decision-making among students analyzing their personal genomes on a whole genome sequencing course: a longitudinal cohort study
Source: Genome Med. 2013 Dec 30;5(12):113. doi: 10.1186/gm518 (PMC3971344; doi:10.1186/gm518)
Supplement: Additional file 2: Figure S2 — Flow chart of entire study. [file gm518-S2.pptx]

## Slide 1
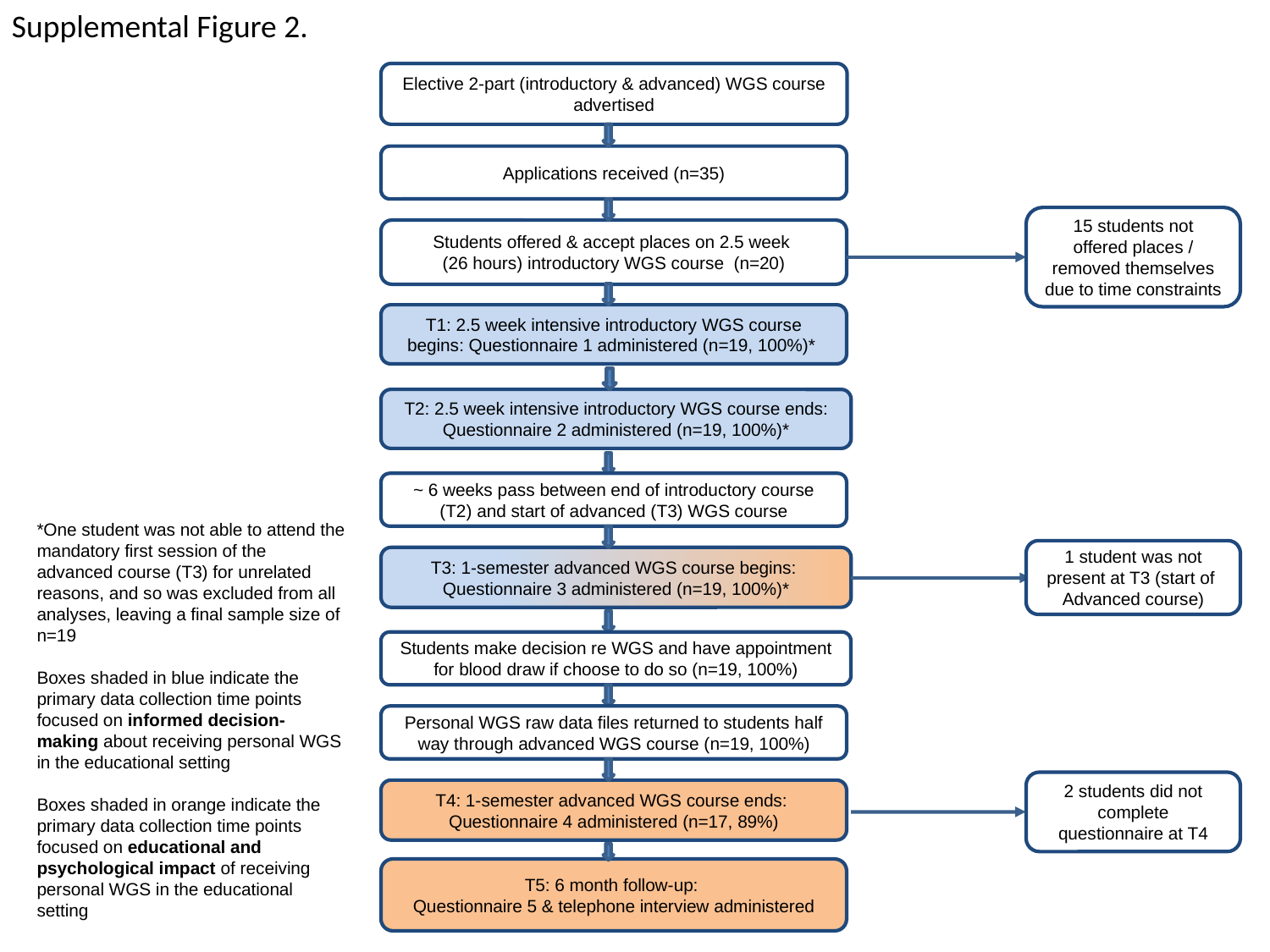

Supplemental Figure 2.
Elective 2-part (introductory & advanced) WGS course advertised
Applications received (n=35)
15 students not offered places / removed themselves due to time constraints
Students offered & accept places on 2.5 week
(26 hours) introductory WGS course (n=20)
T1: 2.5 week intensive introductory WGS course begins: Questionnaire 1 administered (n=19, 100%)*
T2: 2.5 week intensive introductory WGS course ends: Questionnaire 2 administered (n=19, 100%)*
~ 6 weeks pass between end of introductory course (T2) and start of advanced (T3) WGS course
*One student was not able to attend the mandatory first session of the advanced course (T3) for unrelated reasons, and so was excluded from all analyses, leaving a final sample size of n=19
Boxes shaded in blue indicate the primary data collection time points focused on informed decision-making about receiving personal WGS in the educational setting
Boxes shaded in orange indicate the primary data collection time points focused on educational and psychological impact of receiving personal WGS in the educational setting
1 student was not present at T3 (start of Advanced course)
T3: 1-semester advanced WGS course begins:
Questionnaire 3 administered (n=19, 100%)*
Students make decision re WGS and have appointment for blood draw if choose to do so (n=19, 100%)
Personal WGS raw data files returned to students half way through advanced WGS course (n=19, 100%)
2 students did not complete questionnaire at T4
T4: 1-semester advanced WGS course ends:
Questionnaire 4 administered (n=17, 89%)
T5: 6 month follow-up:
Questionnaire 5 & telephone interview administered
