# Supplementary material for: Informed decision-making among students analyzing their personal genomes on a whole genome sequencing course: a longitudinal cohort study
Source: Genome Med. 2013 Dec 30;5(12):113. doi: 10.1186/gm518 (PMC3971344; doi:10.1186/gm518)
Supplement: Additional file 3 — Questionnaire administered before and after the introductory course (T1 and T2). [file gm518-S3.docx]

**Students’ Attitudes Towards the Use of Personal Genome Data in the Classroom**

1. **Decision and decisional conflict**

**We are interested in knowing what your feelings are about analyzing your own versus an anonymous donated genome as part of an advanced whole genome sequencing course in the future. We are interested in knowing what your feelings are about this *at the present time.***

|  | No definitely not | No probably not | Yes probably | Yes definitely | Don’t know | It depends |
| --- | --- | --- | --- | --- | --- | --- |
| Would you want to analyze your own genome as part of an advanced whole genome sequencing course? | □ | □ | □ | □ | □ | □ |

Decisional Conflict Scale (O’Connor et al, 1995)

**At this point, which of the following options would you prefer? Please check one.**

**□** **Option 1:** I would like to analyze my own genome as part of an advanced whole genome sequencing course.

**□** **Option 2:** I would not like to analyze my own genome as part of an advanced whole genome sequencing course, and would rather analyze an anonymous donated genome.

**Considering the option you prefer, please answer the following questions:**

|  | Strongly agree | Agree | Neither agree nor disagree | Disagree | Strongly disagree |
| --- | --- | --- | --- | --- | --- |
| I know which options are available to me. | □ | □ | □ | □ | □ |
| I know the benefits of each option. | □ | □ | □ | □ | □ |
| I know the risks of each option. | □ | □ | □ | □ | □ |
| I am clear about which benefits matter most to me. | □ | □ | □ | □ | □ |
| I am clear about which risks matter most. | □ | □ | □ | □ | □ |
| I am clear about which is more important to me (the benefits or the risks). | □ | □ | □ | □ | □ |
| I have enough support from others to make a choice. | □ | □ | □ | □ | □ |
| I am choosing without pressure from others. | □ | □ | □ | □ | □ |
| I have enough advice to make a choice. | □ | □ | □ | □ | □ |
| I feel sure about what to choose. | □ | □ | □ | □ | □ |
| This decision is easy for me to make. | □ | □ | □ | □ | □ |
| I feel I have made an informed choice. | □ | □ | □ | □ | □ |
| My decision shows what is important to me. | □ | □ | □ | □ | □ |
| I expect to stick with my decision. | □ | □ | □ | □ | □ |
| I am satisfied with my decision. | □ | □ | □ | □ | □ |

Ormond et al (2011) – Stanford study

|  | Strongly disagree [1] | Disagree  [2] | Neither agree nor disagree  [3] | Agree  [4] | Strongly agree  [5] |
| --- | --- | --- | --- | --- | --- |
| I think analyzing my own genome as part of an advanced whole genome sequencing course would be useful | □ | □ | □ | □ | □ |

1. **Reasons for and against using own genome**

Ormond et al (2011) – Stanford study

**Reasons for using own genome:**

|  | Strongly disagree [1] | Disagree  [2] | Neither agree nor disagree  [3] | Agree  [4] | Strongly agree  [5] | Not applicable |
| --- | --- | --- | --- | --- | --- | --- |
| Satisfy general curiosity | □ | □ | □ | □ | □ | □ |
| See if a specific disease runs in the family or is in DNA | □ | □ | □ | □ | □ | □ |
| Learn about genetic makeup without going through a physician | □ | □ | □ | □ | □ | □ |
| Inform family members about health risks | □ | □ | □ | □ | □ | □ |
| Understand what a patient may learn/experience | □ | □ | □ | □ | □ | □ |
| Help understand principles of human genetics | □ | □ | □ | □ | □ | □ |

**Reasons against using own genome:**

|  | Strongly disagree [1] | Disagree  [2] | Neither agree nor disagree  [3] | Agree  [4] | Strongly agree  [5] | Not applicable |
| --- | --- | --- | --- | --- | --- | --- |
| Results are not reliable | □ | □ | □ | □ | □ | □ |
| Results are not accurate | □ | □ | □ | □ | □ | □ |
| Results are not predictive | □ | □ | □ | □ | □ | □ |
| Concern about privacy/risks to privacy | □ | □ | □ | □ | □ | □ |
| Information will not be medically useful/will not change medical decisions | □ | □ | □ | □ | □ | □ |
| Information will not help learn human genetics | □ | □ | □ | □ | □ | □ |
| Unwanted information | □ | □ | □ | □ | □ | □ |
| Costs too much | □ | □ | □ | □ | □ | □ |

1. **Perceived benefits and concerns**

Ormond et al (2011) – Stanford study

**If personal genome sequencing was offered for free as an optional part of an advanced whole genome sequencing class so that I could analyze my results anonymously in relationship to what I was learning with principles discussed generally in class...**

|  | Strongly disagree [1] | Disagree  [2] | Neither agree nor disagree  [3] | Agree  [4] | Strongly agree  [5] | Not applicable |
| --- | --- | --- | --- | --- | --- | --- |
| My own results would help me understand genetics concepts better than someone else’s results. | □ | □ | □ | □ | □ | □ |
| I feel that I would be at a disadvantage to my classmates if I did not undergo the testing. | □ | □ | □ | □ | □ | □ |
| I would see this as an opportunity to get a service that I would not ordinarily get if I had to pay full price. | □ | □ | □ | □ | □ | □ |
| I would be concerned that my professors would know who took up the offer of testing and who did not. | □ | □ | □ | □ | □ | □ |
| I would be concerned that my classmates would know who took up the offer of testing and who did not. | □ | □ | □ | □ | □ | □ |
| I would see this as an opportunity to get information that would help me improve my health. | □ | □ | □ | □ | □ | □ |
| I would be concerned that I might get some results that would be disturbing. | □ | □ | □ | □ | □ | □ |
| I would only take up the offer of testing if I could get genetic counseling before I sent my sample in. | □ | □ | □ | □ | □ | □ |
| I would only take up the offer of testing if I could get genetic counseling after I got my results back in. | □ | □ | □ | □ | □ | □ |
| I would be concerned that people would find out genetic or health information about me. | □ | □ | □ | □ | □ | □ |

1. **General views about whole genome sequencing**

Ormond et al (2011) – Stanford study

|  | **Not useful at all** | **Not very useful** | **Not sure** | **Useful** | **Very useful** | **Not applicable** |
| --- | --- | --- | --- | --- | --- | --- |
| How useful do you think the results from whole genome sequencing will be to a physician? | □ | □ | □ | □ | □ | □ |
| How useful do you think the results from whole genome sequencing information will be to patients themselves? | □ | □ | □ | □ | □ | □ |
|  | **Not at all likely** | **Not very likely** | **Not sure** | **Quite likely** | **Very likely** |  |
| How likely is it that knowing the results from whole genome sequencing for yourself would lead to any changes in your behavior? | □ | □ | □ | □ | □ | □ |
|  | **Strongly disagree** | **Disagree** | **Neither** | **Agree** | **Strongly agree** |  |
| Whole genome sequencing is useful for patients. | □ | □ | □ | □ | □ | □ |
| If I underwent whole genome sequencing, I would ask a physician for help in interpreting the results. | □ | □ | □ | □ | □ | □ |
| Results of whole genome sequencing would influence my future health care decisions. | □ | □ | □ | □ | □ | □ |
| Physicians have a professional responsibility to help individuals understand the results they receive from whole genome sequencing, even if the physician has not ordered the test. | □ | □ | □ | □ | □ | □ |
| Physicians have enough knowledge to help individuals interpret results of whole genome sequencing. | □ | □ | □ | □ | □ | □ |
| Most people can accurately interpret whole genome sequencing results. | □ | □ | □ | □ | □ | □ |
| I know enough about genetics to understand the whole genome sequencing results. | □ | □ | □ | □ | □ | □ |
| I understand the risks and benefits of using getting personal whole genome sequencing done. | □ | □ | □ | □ | □ | □ |

1. **General views about whole genome sequencing**

Adapted from: Ormond et al (2011) – Stanford study

Please read each of the following 3 scenarios, and then answer the questions that follow for each one.

**Scenario 1.**

You have a 37-year-old patient who has a family history of breast and ovarian cancer (her mother with bilateral breast cancer at the age of 45 years, her maternal aunt with ovarian cancer at the age of 52 years, and her maternal grandmother with bilateral breast cancer at the age of 50 years). Because she did not want her insurance company to discriminate against her, she underwent testing through a DTC genetic testing company. She wants you to help her understand her testing results so that she can undergo any appropriate screening and/or prophylactic surgeries.

As epidemiologic background, 13% of the population develops breast cancer in their lifetime, and 5–10% of cases of breast cancer are estimated to be due to a genetic predisposition. The three studies that addressed the SNPs listed below were published in 2007. They are all case-control studies that include between 1,600 –18,290 cases and 4,316 –22,670 controls. The odds ratios ranged between 0.74 and 1.16, depending on the SNP and the study.

Your patient’s results are as follows, and the company interprets this combination of results as a 9% lifetime risk: TNRC9, - +; FGFR2, ++; Chr2.217614077, -+; CASP8 --; MAP3K1, +-; Chr8.128424800, ++; and LSP1, ++.

Presume that + represents the low-risk allele and - represents the at-risk allele.

***What is the best way to interpret your patient’s results? Check as many boxes as apply:***

| Patient is affected with breast cancer | □ |
| --- | --- |
| Patient has higher risk than average | □ |
| Patient has lower risk than average | □ |
| Patient is a carrier of breast cancer and may develop it | □ |
| Patient has no risk for breast cancer | □ |
| A different genetic test should be ordered | □ |
| A different clinical test should be ordered | □ |
| I have no idea what the results mean | □ |

***What issues impacted your understanding of the case? Check as many boxes as apply:***

| Family history | □ |
| --- | --- |
| Samples from the studies | □ |
| Odds ratios from the studies | □ |
| Penetrance of the condition | □ |
| Test results and interpretation by the company | □ |
| None of the above | □ |

***How would you counsel the patient? Check as many boxes as apply:***

| Should have clinical screening for breast cancer | □ |
| --- | --- |
| Not at increased risk but should let family know they are a carrier and others may be at risk | □ |
| Not at increased risk and no additional intervention needed | □ |
| I have no idea how to counsel the patient | □ |

**Scenario 2.**

Your patient comes to see you with the results from their genomic testing through a DTC genetic testing company. You see that they have undergone genetic testing for hemochromatosis. Through your research on websites like OMIM you learn that hemochromatosis is a condition that is inherited in an autosomal recessive manner with decreased penetrance (estimates vary from 1 to 10% depending on the specific mutation). You also learn that the treatments for hemochromatosis are regular phlebotomy to reduce the chance for clinical complications due to iron overload.

The results are as follows: HFE-C282Y, ++; HFE-H63D, +-; and HFE-S65C, ++.

Presume that + represents the low-risk allele and - represents the at-risk allele.

***What is the best way to interpret your patient’s results? Check as many boxes as apply:***

| Patient is affected with hemochromatosis | □ |
| --- | --- |
| Patient has higher risk than average | □ |
| Patient has lower risk than average | □ |
| Patient is a carrier of hemochromatosis and may develop it | □ |
| Patient has no risk for hemochromatosis | □ |
| A different genetic test should be ordered | □ |
| A different clinical test should be ordered | □ |
| I have no idea what the results mean | □ |

***What issues impacted your understanding of the case? Check as many boxes as apply:***

| Mode of inheritance | □ |
| --- | --- |
| Penetrance of the condition | □ |
| Test results and interpretation by the company |  |
| None of the above | □ |

***How would you counsel the patient? Check as many boxes as apply:***

| Should have clinical screening for hemochromatosis | □ |
| --- | --- |
| Not at increased risk but should let family know they are a carrier and others may be at risk | □ |
| Not at increased risk and no additional intervention needed | □ |
| I have no idea how to counsel the patient | □ |

**Scenario 3.**

Your patient has a grandparent with macular degeneration. He is concerned about the chance he may develop it. About 3% of the population develops macular degeneration, and you learn that about 66% of the risk for macular degeneration is due to a genetic predisposition. You also learn that one of the studies that influenced available genetic testing included 4757 phenotyped subjects, but that not all of them had genetic testing results.

You review their genetic testing results and find the following: LOC387715-S69A, -+; CFH-intron, --; CFB, --; C2-E318D, --; CFH-Y402H, -+; and C3-R80G, --.

Presume that - represents the low-risk allele and + represents the at-risk allele.

***What is the best way to interpret your patient’s results? Check as many boxes as apply:***

| Patient is affected with macular degeneration | □ |
| --- | --- |
| Patient has higher risk than average | □ |
| Patient has lower risk than average | □ |
| Patient is a carrier of macular degeneration and may develop it | □ |
| Patient has no risk for macular degeneration | □ |
| A different genetic test should be ordered | □ |
| A different clinical test should be ordered | □ |
| I have no idea what the results mean | □ |

***What issues impacted your understanding of the case? Check as many boxes as apply:***

| Family history |  |
| --- | --- |
| Study sizes |  |
| Samples from the studies |  |
| Odds ratios from the studies | □ |
| Penetrance of the condition | □ |
| Test results and interpretation by the company |  |
| None of the above | □ |

***How would you counsel the patient? Check as many boxes as apply:***

| Should have clinical screening for macular degeneration | □ |
| --- | --- |
| Not at increased risk but should let family know they are a carrier and others may be at risk | □ |
| Not at increased risk and no additional intervention needed | □ |
| I have no idea how to counsel the patient | □ |

**Finally, we are very interested in any additional thoughts or comments you might have regarding the possibility of analyzing personal genomes in an advanced whole genome sequencing course. Please write any suggestions, comments, concerns, thoughts or questions in the box below.**

***Thank you very much for taking the time to complete this questionnaire!***
