# Supplementary material for: Informed decision-making among students analyzing their personal genomes on a whole genome sequencing course: a longitudinal cohort study
Source: Genome Med. 2013 Dec 30;5(12):113. doi: 10.1186/gm518 (PMC3971344; doi:10.1186/gm518)
Supplement: Additional file 4 — Questionnaire administered at the start of the advanced course (T3). [file gm518-S4.docx]

**Personal Genome Analysis in the Classroom**

**PRECOURSE QUESTIONNAIRE**

1. **Decision and decisional conflict**

**We are interested in knowing what your feelings are about analyzing your own versus an anonymous donated genome as part of this whole genome sequencing course. We are interested in knowing what your feelings are about this *at the present time.***

|  | No definitely not | No probably not | Yes probably | Yes definitely | Don’t know | It depends |
| --- | --- | --- | --- | --- | --- | --- |
| Would you want to analyze your own genome as part of this whole genome sequencing course? | □ | □ | □ | □ | □ | □ |

Decisional Conflict Scale (O’Connor et al, 1995)

**At this point, which of the following options would you prefer? Please check one.**

**□** **Option 1:** I would like to analyze my own genome as part of this whole genome sequencing course.

**□** **Option 2:** I would not like to analyze my own genome as part of this whole genome sequencing course, and would rather analyze an anonymous donated genome.

**Considering the option you prefer, please answer the following questions:**

|  | Strongly agree | Agree | Neither agree nor disagree | Disagree | Strongly disagree |
| --- | --- | --- | --- | --- | --- |
| I know which options are available to me. | □ | □ | □ | □ | □ |
| I know the benefits of each option. | □ | □ | □ | □ | □ |
| I know the risks of each option. | □ | □ | □ | □ | □ |
| I am clear about which benefits matter most to me. | □ | □ | □ | □ | □ |
| I am clear about which risks matter most. | □ | □ | □ | □ | □ |
| I am clear about which is more important to me (the benefits or the risks). | □ | □ | □ | □ | □ |
| I have enough support from others to make a choice. | □ | □ | □ | □ | □ |
| I am choosing without pressure from others. | □ | □ | □ | □ | □ |
| I have enough advice to make a choice. | □ | □ | □ | □ | □ |
| I feel sure about what to choose. | □ | □ | □ | □ | □ |
| This decision is easy for me to make. | □ | □ | □ | □ | □ |
| I feel I have made an informed choice. | □ | □ | □ | □ | □ |
| My decision shows what is important to me. | □ | □ | □ | □ | □ |
| I expect to stick with my decision. | □ | □ | □ | □ | □ |
| I am satisfied with my decision. | □ | □ | □ | □ | □ |

Ormond et al (2011) – Stanford study

|  | Strongly disagree [1] | Disagree  [2] | Neither agree nor disagree  [3] | Agree  [4] | Strongly agree  [5] |
| --- | --- | --- | --- | --- | --- |
| I think analyzing my own genome as part of this whole genome sequencing course would be useful | □ | □ | □ | □ | □ |

|  | No definitely not | No probably not | Yes probably | Yes definitely | Don’t know | It depends |
| --- | --- | --- | --- | --- | --- | --- |
| I intend to have the blood draw for whole genome sequencing as part of this course | □ | □ | □ | □ | □ | □ |

|  | No definitely not | No probably not | Yes probably | Yes definitely | Don’t know | It depends |
| --- | --- | --- | --- | --- | --- | --- |
| I intend to analyze my own genome as part of this whole genome sequencing course | □ | □ | □ | □ | □ | □ |

|  | No definitely not | No probably not | Yes probably | Yes definitely | Don’t know | It depends |
| --- | --- | --- | --- | --- | --- | --- |
| I intend to seek genetic counseling before making my decision about whether to obtain my own personal genome sequence data | □ | □ | □ | □ | □ | □ |

1. **Reasons for and against using own genome**

Ormond et al (2011) – Stanford study

**Reasons for using own genome:**

|  | Strongly disagree [1] | Disagree  [2] | Neither agree nor disagree  [3] | Agree  [4] | Strongly agree  [5] | Not applicable |
| --- | --- | --- | --- | --- | --- | --- |
| Satisfy general curiosity | □ | □ | □ | □ | □ | □ |
| See if a specific disease runs in the family or is in DNA | □ | □ | □ | □ | □ | □ |
| Learn about genetic makeup without going through a physician | □ | □ | □ | □ | □ | □ |
| Inform family members about health risks | □ | □ | □ | □ | □ | □ |
| Understand what a patient may learn/experience | □ | □ | □ | □ | □ | □ |
| Help understand principles of human genetics | □ | □ | □ | □ | □ | □ |
| Other (please specify): | | | | | | |

**Reasons against using own genome:**

|  | Strongly disagree [1] | Disagree  [2] | Neither agree nor disagree  [3] | Agree  [4] | Strongly agree  [5] | Not applicable |
| --- | --- | --- | --- | --- | --- | --- |
| Results are not reliable | □ | □ | □ | □ | □ | □ |
| Results are not accurate | □ | □ | □ | □ | □ | □ |
| Results are not predictive | □ | □ | □ | □ | □ | □ |
| Concern about privacy/risks to privacy | □ | □ | □ | □ | □ | □ |
| Information will not be medically useful/will not change medical decisions | □ | □ | □ | □ | □ | □ |
| Information will not help learn human genetics | □ | □ | □ | □ | □ | □ |
| Unwanted information | □ | □ | □ | □ | □ | □ |
| Costs too much | □ | □ | □ | □ | □ | □ |
| Other (please specify): | | | | | | |

**Have you discussed whether or not to get your genome sequenced as part of this course with anyone?**

| Yes | No |
| --- | --- |
| □ | □ |

**Who have you talked to about whether or not to get your genome sequenced as part of this course? (check all that apply)**

| Genetic counselor | Other health professional |  |  |  |
| --- | --- | --- | --- | --- |
| □ | □ |  |  |  |
| Mother | Father | Brother/sister | Other family member |  |
| □ | □ | □ | □ |  |
| Friend(s) | Spouse/significant other | One or more of the course directors | Other | If other, please specify: |
| □ | □ | □ | □ | _____________________ |
|  |  |  |  | _____________________ |

1. **Perceived benefits and concerns**

Ormond et al (2011) – Stanford study

**If personal genome sequencing is offered for free as an optional part of this whole genome sequencing class...**

|  | Strongly disagree [1] | Disagree  [2] | Neither agree nor disagree  [3] | Agree  [4] | Strongly agree  [5] | Not applicable |
| --- | --- | --- | --- | --- | --- | --- |
| My own results would help me understand genetics concepts better than someone else’s results. | □ | □ | □ | □ | □ | □ |
| I feel that I would be at a disadvantage to my classmates if I did not undergo the testing. | □ | □ | □ | □ | □ | □ |
| I would see this as an opportunity to get a service that I would not ordinarily get if I had to pay full price. | □ | □ | □ | □ | □ | □ |
| I would be concerned that my professors would know who took up the offer of testing and who did not. | □ | □ | □ | □ | □ | □ |
| I would be concerned that my classmates would know who took up the offer of testing and who did not. | □ | □ | □ | □ | □ | □ |
| I would see this as an opportunity to get information that would help me improve my health. | □ | □ | □ | □ | □ | □ |
| I would be concerned that I might get some results that would be disturbing. | □ | □ | □ | □ | □ | □ |
| I would only take up the offer of testing if I could get genetic counseling before I sent my sample in. | □ | □ | □ | □ | □ | □ |
| I would only take up the offer of testing if I could get genetic counseling after I got my results back in. | □ | □ | □ | □ | □ | □ |
| I would be concerned that people would find out genetic or health information about me. | □ | □ | □ | □ | □ | □ |

1. **General views about whole genome sequencing**

Ormond et al (2011) – Stanford study

|  | **Not useful at all** | **Not very useful** | **Not sure** | **Useful** | **Very useful** | **Not applicable** |
| --- | --- | --- | --- | --- | --- | --- |
| How useful do you think the results from whole genome sequencing will be to a physician? | □ | □ | □ | □ | □ | □ |
| How useful do you think the results from whole genome sequencing information will be to patients themselves? | □ | □ | □ | □ | □ | □ |
|  | **Not at all likely** | **Not very likely** | **Not sure** | **Quite likely** | **Very likely** |  |
| How likely is it that knowing the results from whole genome sequencing for yourself would lead to any changes in your behavior? | □ | □ | □ | □ | □ | □ |
|  | **Strongly disagree** | **Disagree** | **Neither** | **Agree** | **Strongly agree** |  |
| Whole genome sequencing is useful for patients. | □ | □ | □ | □ | □ | □ |
| If I underwent whole genome sequencing, I would ask a physician for help in interpreting the results. | □ | □ | □ | □ | □ | □ |
| Results of whole genome sequencing would influence my future health care decisions. | □ | □ | □ | □ | □ | □ |
| Physicians have a professional responsibility to help individuals understand the results they receive from whole genome sequencing, even if the physician has not ordered the test. | □ | □ | □ | □ | □ | □ |
| Physicians have enough knowledge to help individuals interpret results of whole genome sequencing. | □ | □ | □ | □ | □ | □ |
| Most people can accurately interpret whole genome sequencing results. | □ | □ | □ | □ | □ | □ |
| I know enough about genetics to understand the whole genome sequencing results. | □ | □ | □ | □ | □ | □ |
| I understand the risks and benefits of using getting personal whole genome sequencing done. | □ | □ | □ | □ | □ | □ |

1. **Anxiety**

STAI (Speilberger, 1968)

A number of statements which people have used to describe themselves are given below. Read each statement and then circle the appropriate number to the right of the statement to indicate how you feel *right* now, that is, *at this moment*. There are no right or wrong answers. Do not spend too much time on any one statement but give the answer which seems to describe your present feelings best.

|  | **Not at all**  **(1)** | **Somewhat**  **(2)** | **Moderately so (3)** | **Very much so (4)** |
| --- | --- | --- | --- | --- |
| 1. I feel calm | □ | □ | □ | □ |
| 2. I feel secure | □ | □ | □ | □ |
| 3. I am tense | □ | □ | □ | □ |
| 4. I feel strained | □ | □ | □ | □ |
| 5. I feel at ease | □ | □ | □ | □ |
| 6. I feel upset | □ | □ | □ | □ |
| 7. I am presently worrying over possible misfortunes | □ | □ | □ | □ |
| 8. I feel satisfied | □ | □ | □ | □ |
| 9. I feel frightened | □ | □ | □ | □ |
| 10. I feel comfortable | □ | □ | □ | □ |
| 11. I feel self-confident | □ | □ | □ | □ |
| 12. I feel nervous | □ | □ | □ | □ |
| 13. I am jittery | □ | □ | □ | □ |
| 14. I feel indecisive | □ | □ | □ | □ |
| 15. I am relaxed | □ | □ | □ | □ |
| 16. I feel content | □ | □ | □ | □ |
| 17. I am worried | □ | □ | □ | □ |
| 18. I feel confused | □ | □ | □ | □ |
| 19. I feel steady | □ | □ | □ | □ |
| 20. I feel pleasant | □ | □ | □ | □ |

1. **Depression**

Center for Epidemiologic Studies Depression Scale (CES-D)

Below is a list of the ways you might have felt or behaved. Please indicate how often you have felt this way during the past week.

|  | **Rarely or none of the time (less than 1 day)** | **Some or a little of the time (1-2 days)** | **Occasionally or a moderate amount of time (3-4 days)** | **Most or all of the time (5-7 days)** |
| --- | --- | --- | --- | --- |
| 1. I was bothered by things that usually don’t bother me. | □ | □ | □ | □ |
| 2. I did not feel like eating; my appetite was poor. | □ | □ | □ | □ |
| 3. I felt that I could not shake off the blues even with help from my family or friends. | □ | □ | □ | □ |
| 4. I felt I was just as good as other people. | □ | □ | □ | □ |
| 5. I had trouble keeping my mind on what I was doing. | □ | □ | □ | □ |
| 6. I felt depressed. | □ | □ | □ | □ |
| 7. I felt that everything I did was an effort. | □ | □ | □ | □ |
| 8. I felt hopeful about the future. | □ | □ | □ | □ |
| 9. I thought my life had been a failure. | □ | □ | □ | □ |
| 10. I felt fearful. | □ | □ | □ | □ |
| 11. My sleep was restless. | □ | □ | □ | □ |
| 12. I was happy. | □ | □ | □ | □ |
| 13. I talked less than usual. | □ | □ | □ | □ |
| 14. I felt lonely. | □ | □ | □ | □ |
| 15. People were unfriendly. | □ | □ | □ | □ |
| 16. I enjoyed life. | □ | □ | □ | □ |
| 17. I had crying spells. | □ | □ | □ | □ |
| 18. I felt sad. | □ | □ | □ | □ |
| 19. I felt that people dislike me. | □ | □ | □ | □ |
| 20. I could not get “going.” | □ | □ | □ | □ |

1. **Subjective understanding & self-efficacy**

Healthy Subjects Study

**How would you describe your current understanding of genetics?**

| None | Minimal | Some | Moderate | High |
| --- | --- | --- | --- | --- |
| □ | □ | □ | □ | □ |

**How would you rate your knowledge of genetics compared with others?**

| Much less  than others | Less than others | As much as others | More than others | Much more  than others |
| --- | --- | --- | --- | --- |
| □ | □ | □ | □ | □ |

**How would you describe your current understanding of whole genome sequencing?**

| None | Minimal | Some | Moderate | High |
| --- | --- | --- | --- | --- |
| □ | □ | □ | □ | □ |

**How would you rate your knowledge of whole genome sequencing compared with others?**

| Much less  than others | Less than others | As much as others | More than others | Much more  than others |
| --- | --- | --- | --- | --- |
| □ | □ | □ | □ | □ |

New

**How confident are you in your ability to analyze and interpret whole genome sequence data?**

| None | Minimal | Some | Moderate | High |
| --- | --- | --- | --- | --- |
| □ | □ | □ | □ | □ |

1. **Knowledge about whole genome sequencing**

O 2011

Please read each of the following 3 scenarios, and then answer the questions that follow for each one.

**Scenario 1.**

You have a 37-year-old patient who has a family history of breast and ovarian cancer (her mother with bilateral breast cancer at the age of 45 years, her maternal aunt with ovarian cancer at the age of 52 years, and her maternal grandmother with bilateral breast cancer at the age of 50 years). Because she did not want her insurance company to discriminate against her, she underwent testing through a DTC genetic testing company. She wants you to help her understand her testing results so that she can undergo any appropriate screening and/or prophylactic surgeries.

As epidemiologic background, 13% of the population develops breast cancer in their lifetime, and 5–10% of cases of breast cancer are estimated to be due to a genetic predisposition. The three studies that addressed the SNPs listed below were published in 2007. They are all case-control studies that include between 1,600 –18,290 cases and 4,316 –22,670 controls. The odds ratios ranged between 0.74 and 1.16, depending on the SNP and the study.

Your patient’s results are as follows, and the company interprets this combination of results as a 9% lifetime risk: TNRC9, - +; FGFR2, ++; Chr2.217614077, -+; CASP8 --; MAP3K1, +-; Chr8.128424800, ++; and LSP1, ++.

Presume that + represents the low-risk allele and - represents the at-risk allele.

**Q1. What is the best way to interpret your patient’s results? Check as many boxes as apply:**

| Patient is affected with breast cancer | □ |
| --- | --- |
| Patient has higher risk than average | □ |
| Patient has lower risk than average | □ |
| Patient is a carrier of breast cancer and may develop it | □ |
| Patient has no risk for breast cancer | □ |
| A different genetic test should be ordered | □ |
| A different clinical test should be ordered | □ |
| I have no idea what the results mean | □ |

**Q2. What issues impacted your understanding of the case? Check as many boxes as apply:**

| Family history | □ |
| --- | --- |
| Samples from the studies | □ |
| Odds ratios from the studies | □ |
| Penetrance of the condition | □ |
| Test results and interpretation by the company | □ |
| None of the above | □ |

**Q3. How would you counsel the patient? Check as many boxes as apply:**

| Should have clinical screening for breast cancer | □ |
| --- | --- |
| Not at increased risk but should let family know they are a carrier and others may be at risk | □ |
| Not at increased risk and no additional intervention needed | □ |
| I have no idea how to counsel the patient | □ |

**Scenario 2.**

Your patient comes to see you with the results from their genomic testing through a DTC genetic testing company. You see that they have undergone genetic testing for hemochromatosis. Through your research on websites like OMIM you learn that hemochromatosis is a condition that is inherited in an autosomal recessive manner with decreased penetrance (estimates vary from 1 to 10% depending on the specific mutation). You also learn that the treatments for hemochromatosis are regular phlebotomy to reduce the chance for clinical complications due to iron overload.

The results are as follows: HFE-C282Y, ++; HFE-H63D, +-; and HFE-S65C, ++.

Presume that + represents the low-risk allele and - represents the at-risk allele.

**Q4. What is the best way to interpret your patient’s results? Check as many boxes as apply:**

| Patient is affected with hemochromatosis | □ |
| --- | --- |
| Patient has higher risk than average | □ |
| Patient has lower risk than average | □ |
| Patient is a carrier of hemochromatosis and may develop it | □ |
| Patient has no risk for hemochromatosis | □ |
| A different genetic test should be ordered | □ |
| A different clinical test should be ordered | □ |
| I have no idea what the results mean | □ |

**Q5. What issues impacted your understanding of the case? Check as many boxes as apply:**

| Mode of inheritance | □ |
| --- | --- |
| Penetrance of the condition | □ |
| Test results and interpretation by the company |  |
| None of the above | □ |

**Q6. How would you counsel the patient? Check as many boxes as apply:**

| Should have clinical screening for hemochromatosis | □ |
| --- | --- |
| Not at increased risk but should let family know they are a carrier and others may be at risk | □ |
| Not at increased risk and no additional intervention needed | □ |
| I have no idea how to counsel the patient | □ |

**Scenario 3.**

Your patient has a grandparent with macular degeneration. He is concerned about the chance he may develop it. About 3% of the population develops macular degeneration, and you learn that about 66% of the risk for macular degeneration is due to a genetic predisposition. You also learn that one of the studies that influenced available genetic testing included 4757 phenotyped subjects, but that not all of them had genetic testing results.

You review their genetic testing results and find the following: LOC387715-S69A, -+; CFH-intron, --; CFB, --; C2-E318D, --; CFH-Y402H, -+; and C3-R80G, --.

Presume that - represents the low-risk allele and + represents the at-risk allele.

**Q7. What is the best way to interpret your patient’s results? Check as many boxes as apply:**

| Patient is affected with macular degeneration | □ |
| --- | --- |
| Patient has higher risk than average | □ |
| Patient has lower risk than average | □ |
| Patient is a carrier of macular degeneration and may develop it | □ |
| Patient has no risk for macular degeneration | □ |
| A different genetic test should be ordered | □ |
| A different clinical test should be ordered | □ |
| I have no idea what the results mean | □ |

**Q8. What issues impacted your understanding of the case? Check as many boxes as apply:**

| Family history |  |
| --- | --- |
| Study sizes |  |
| Samples from the studies |  |
| Odds ratios from the studies | □ |
| Penetrance of the condition | □ |
| Test results and interpretation by the company |  |
| None of the above | □ |

**Q9. How would you counsel the patient? Check as many boxes as apply:**

| Should have clinical screening for macular degeneration | □ |
| --- | --- |
| Not at increased risk but should let family know they are a carrier and others may be at risk | □ |
| Not at increased risk and no additional intervention needed | □ |
| I have no idea how to counsel the patient | □ |

JD

**Q10. An individual is found to have a novel variant in the protein coding region of a gene which causes truncation (i.e. shortening) of the encoded protein sequence. Is the presence of this variant certain to lead to disease or other abnormal physiology?**

Yes □

No □

**Q11. True or False: Causal disease and pharmacogenomic mutations are only found in the protein coding region of the genome.**

True □

False □

**Q12. True or False: A SNP loci is associated with Type 2 diabetes at genome-wide significance levels in large studies of multiple native European and Asian populations, therefore it is likely to be broadly predictive of Type 2 diabetes risk irrespective of ethnicity.**

True □

False □

AB

**Q13. You are presented with a human genome sequenced at 2X coverage. For simplification assume that all sequenced reads could be mapped to their correct location on the genome (i.e. ignore sequence quality, repeats, variation, etc.). According to Lander-Waterman statistics, approximately what fraction of the genome do you expect NOT to be covered by at least one read? Choose the closest percentage.**

□ a) 5%

□ b) 15%

□ c) 25%

□ d) 35%

□ e) 45%

**Q14. You are examining the mapping of reads within a sam file. You come across the following mapping information for a read. What is the best description of this read?**

HWI-ST276:194:D08CTACXX:1:1101:21082:2161 16 chr20 37452491 37 73M1D27M * 0 0 TAGATTTCATTTATTTTCCTTCAAGG

ACTCCTCTTACCGTCATGCCTCCGTGGCTCATCTTTCCTGTCTGACATTTTTTTTTTATTTTTAAGGAGAGGGG :CACCDDCCCCCCCDCBDCAACCC@C??8BA:CBB=DBCCDDDBDBAFFGHHHIIIGBGGIHEGDGHGIIHIIJ

□ a) High mapping quality, with no variation to reference

□ b) Low mapping quality, with no variation relative to reference

□ c) High mapping quality, with variation relative to reference

□ d) Low mapping quality, with variation relative to reference

□ e) Unmapped

**Q15. You are examining a VCF file of an individual sequenced at 30X coverage and come across the following interesting variants:**

**1)**

**chr1 11854457 rs4846051 G A 1003.49 <MASKED> AC=2;AF=1.00;AN=2;DB;DP=31;Dels=0.00;FS=0.000;HRun=2;HaplotypeScore=0.0000;MQ=59.25;MQ0=0;QD=32.37;SB=-287.97;SNPEFF_AMINO_ACID_CHANGE=F435;SNPEFF_CODON_CHANGE=ttC/ttT;SNPEFF_EFFECT=SYNONYMOUS_CODING;SNPEFF_EXON_ID=NM_005957.ex.5;SNPEFF_FUNCTIONAL_CLASS=SILENT;SNPEFF_GENE_BIOTYPE=mRNA;SNPEFF_GENE_NAME=MTHFR;SNPEFF_IMPACT=LOW;SNPEFF_TRANSCRIPT_ID=NM_005957;VQSLOD=11.4398;culprit=FS GT:AD:DP:GQ:PL 1/1:0,31:31:87.26:1036,87,0**

**2)**

**chr1 26211207 rs113889629 A G 315.68 <MASKED> AC=2;AF=1.00;AN=2;DB;DP=20;Dels=0.00;FS=0.000;HRun=0;HaplotypeScore=2.8995;MQ=46.67;MQ0=1;QD=15.78;SB=-0.01;SNPEFF_EFFECT=UTR_3_PRIME;SNPEFF_FUNCTIONAL_CLASS=NONE;SNPEFF_GENE_BIOTYPE=mRNA;SNPEFF_GENE_NAME=STMN1;SNPEFF_IMPACT=MODIFIER;SNPEFF_TRANSCRIPT_ID=NM_001145454;VQSLOD=3.0717;culprit=MQ GT:AD:DP:GQ:PL 1/1:4,16:20:27.09:349,27,0**

**3) chr1 13183225 rs28634306 T C 1297.32 <MASKED> AC=1;AF=0.50;AN=2;BaseQRankSum=10.122;DB;DP=173;Dels=0.00;FS=164.230;HRun=0;HaplotypeScore=6.0729;MQ**

**=39.28;MQ0=6;MQRankSum=-7.124;QD=7.50;ReadPosRankSum=-1.245;SB=-0.01;SNPEFF_AMINO_ACID_CHANGE=K216;SNPEFF_CODON_CHANGE=aaA/aaG;SNPEFF_EFFECT=SYNONYMOUS_CODING;SNPEFF_EXON_ID=NM_001136561.ex.1;SNPEFF_FUNCT**

**IONAL_CLASS=SILENT;SNPEFF_GENE_BIOTYPE=mRNA;SNPEFF_GENE_NAME=LOC440563;SNPEFF_IMPACT=LOW;SNPEFF_TRANSCRIPT_ID=NM_001136561;VQSLOD=-202.9017;culprit=FS GT:AD:DP:GQ:PL 0/1:114,59:173:99:1327,0,3222**

**c**

**What is the relative order you would place these in, based purely on the mapping and variant quality call information. Note that the passing filter information has been masked from each read.**

□ a) 1,2,3 □ b) 1,3,2 □ c) 2,1,3

□ d) 2,3,1 □ e) 3,1,1 □ f) 3,2,1

SS

**Q16. A 65-year old male is being scheduled for a stent placement. You order a rapid turnaround *CYP2C19* genotyping test from a CLIA-certified laboratory to guide anti-platelet therapy and the result is **1/*2*. Which of the following is the most appropriate interpretation of these results?**

□ A. This patient is an ultra-rapid metabolizer and, therefore, a decreased dose of clopidogrel (Plavix^®^) should be considered.

□ B. This patient is an extensive (normal) metabolizer and, therefore, a normal dose of clopidogrel (Plavix^®^) should be considered.

□ C. This patient is an intermediate metabolizer and, therefore, an increased dose of clopidogrel (Plavix^®^) or an alternative anti-platelet agent should be considered.

□ D. This patient is a poor metabolizer and, therefore, an increased dose of clopidogrel (Plavix^®^) or an alternative anti-platelet agent should be considered.

□ E. It is not possible to interpret these results without additional information.

□ F. I don’t know the most appropriate interpretation of these results.

**Q17. Which of the following enzymes is not known to be involved in the metabolism of human therapeutics?**

□ A. CYP2C9.

□ B. CYP3A4.

□ C. CYP27C1.

□ D. VKORC1.

□ E. CYP2D6.

□ F. CYP2C19

□ G. I don’t know which enzyme is the correct answer.

**Q18. A 70-year old African-American male with new onset atrial fibrillation will require warfarin (Coumadin^®^) therapy to reach a target international normalized ratio (INR) of 2-3. The patient is nervous because his sister previously had a major bleed while on warfarin. He is not a smoker and does not have liver disease. He has not begun warfarin therapy yet as he is currently on lovenox, and he does not take any other medications. His cardiologist would like to use the patient’s genotype to get an estimate of the expected therapeutic warfarin dose. Which of the following genes are the most appropriate to test?**

□ A. *CYP2C19* and *VKORC1*.

□ B. *CYP2C9* and *VKORC1*.

□ C. *CYP2D6* and *VKORC1*.

□ D. Only *VKORC1*.

□ E. I don’t know the most appropriate genes to test for warfarin dose prediction.

ML (S 210)

**Q19. Recent research into the genetics of height estimates a heritability of roughly 80%, but so far GWAS have only been able to explain 5% of the phenotypic variation.**

**Q19. (a) List 3 possible explanations for the missing heritability of complex diseases from traditional GWAS. Assume the studies use conventional DNA microarrays containing tag SNPs for common polymorphisms and established statistical testing methodologies.**

**Q19. (b) For the explanations in part (a), propose an experiment or method of analysis that could go beyond the results obtained from a standard GWAS.**

**Q20. The following two SNPs were shown to be associated with risk for type 2 diabetes in two GWAS studies.**

| **snp** | **odds ratio** | **p-value** | **cases** | **controls** |
| --- | --- | --- | --- | --- |
| rs4402960 | 1.14 | 8.9 x 10^-16^ | 14586 | 17968 |
| rs7754840 | 1.28 | 3.5x10^-7^ | 1921 | 1622 |

**Q20. (a)** **Which SNP has a larger effect size on risk for type 2 diabetes? ________**

**Q20. (b) Which SNP is most statistically significant for risk for type 2 diabetes; i.e. which SNP is most likely to have a true association? ________**

**Q20. (c)  Is the SNP with the biggest effect size on risk for type 2 diabetes always going to be the SNP that is most statistically significant? _________**

**Q20. (d) rs7754840 is a SNP that lies within the CDKAL1 gene.  This SNP was identified because it was contained on the Illumina Chip used for genotyping in the GWAS study.**

**Does this result indicate that rs7754840 is the causal mutation? ________**

**Does this result indicate that CDKAL1 is involved in type 2 diabetes? ________**

**Q21. Two parents are considering having another child. They have the following genotypes at disease associated SNPs.**

| SNP | Phenotype | Risk Allele/Genotype | Mother | Father | Notes |
| --- | --- | --- | --- | --- | --- |
| rs1800562 | hemochromatosis | A | AG | GG | Of AA homozygotes, 30% of males had iron-overload related disease, 1% if females did |
| rs77944974 | Breast Cancer, BRCA1 | DD | DI | II | Lifetime risk of breast cancer for women is increased from 12% to about 60% and risk of ovarian cancer is increased from less than 2% to about 40%. |
| rs113993960 | Cystic Fibrosis | DD | DI | DI |  |
| rs334 | Sickle Cell Anemia | TT | AA | AA |  |

**What is the chance that their child will have that disease?**

**Q21. (a) Chance of hemochromatosis: __________**

**Q21. (b) Chance of breast cancer (BRCA1): __________**

**Q22. (c) Chance of cystic fibrosis: __________**

**Q23. (d) Chance of sickle cell anemia: __________**

**Finally, we are very interested in any additional thoughts or comments you might have regarding having the option to analyzing your personal genome in this whole genome sequencing course, or any other aspect of the course or this research questionnaire. Please write any suggestions, comments, concerns, thoughts or questions in the box below.**

***Thank you very much for taking the time to complete this questionnaire!***
