# Supplementary material for: Informed decision-making among students analyzing their personal genomes on a whole genome sequencing course: a longitudinal cohort study
Source: Genome Med. 2013 Dec 30;5(12):113. doi: 10.1186/gm518 (PMC3971344; doi:10.1186/gm518)
Supplement: Additional file 5: Table S1 — Quantitative measures assessed in entire study. Table S2: Students’ interest in analyzing their own genomes in the educational setting and uncertainty (conflict) around that decision at T1, T2 and T3. Table S3: Students’ attitudes towards personal whole genome sequencing in general. Table S4: Students’ knowledge about personal genomic results based on three clinical scenarios. Table S5: Students’ responses to the open-ended questions comments sections of the questionnaires. [file gm518-S5.pdf]

Supplemental Table 1. Quantitative measures assessed in entire study

Questionnaire measure	Source of measure	T1	T2	T3	T4	T5
**Informed decision-making variables**						
Interest in analyzing own genome in classroom	O'Connor et al (1995)	X	X	X	(x)	-
Decisional conflict (or 'decision uncertainty')	O'Connor et al (1995)	X	X	X	(x)	-
Attitudes/values re analyzing own genome in classroom	Ormond et al (2011)	X	X	X	(x)	-
General attitudes re whole genome sequencing (WGS)	Ormond et al (2011)	X	X	X	(x)	-
Knowledge regarding genomic results scenarios	Ormond et al (2011)	X	X	X	(x)	-
Discussed WGS decision with others	New	-	-	X	(x)	-
**Educational and psychological impact variables**						
Knowledge: extensive test questions re WGS	New (Sinai course directors)	-	-	X	X	-
Subjective understanding of genetics & WGS	MSSM Healthy Subjects	-	-	X	X	X
Confidence in ability to analyze & interpret WGS data	New	-	-	X	X	X
Anxiety (State)	STAI: Speilberger (1968)	-	-	X	X	X
Depression	CES-D	-	-	X	X	X
Actual decision to get WGS	New	-	-	-	-	X
Discussed / shared WGS with others	New	-	-	-	-	X
Decision regret	Brehaut et al (2002)	-	-	-	-	X
Satisfaction with decision	SDS: Holmes-Rovner et al (1995)	-	-	-	-	X
Discussed / will discuss with others post-WGS	New	-	-	-	-	X
Perceived usefulness of having had WGS (1 item)	Ormond et al (2011)	-	-	-	-	X
WGS results obtained (important; specific)	New	-	-	-	-	X
Psychological impact of WGS results	MICRA: Lerman et al (2002)	-	-	-	-	X
Impact of WGS info on family	New	-	-	-	-	X
Educational impact of WGS info	New	-	-	-	-	X
Additional comments	N/A	X	X	X	X	X

Supplemental Table 2. Students' attitudes towards personal whole genome sequencing in the educational setting

N = 19	Response Options	T1	T2	T3	Significance [a]		
**Perceived usefulness of educational WGS**		**N(%)**	**N(%)**	**N(%)**	**T1-T2†**	**T1-T3†**	**T2-T3†**
I think analyzing my own genome as part of an advanced WGS course would be useful [a]	Strongly disagree	1(5.3%)	1(5.3%)	0(0%)	z=-1.34, p=0.18, r=-0.31	z=-1.39, p=0.17, r=0.32	z=-0.63, p=0.53, r=0.14
	Disagree	0(0%)	0(0%)	0(0%)			
	Neither agree nor disagree	2(10.5%)	1(5.3%)	0(0%)			
	Agree	10(52.6%)	9(47.4%)	11(57.9%)			
	Strongly agree	6(31.6%)	8(42.1%)	8(42.1%)			
**Reasons *for* using own genome [b] (agree / strongly agree)**		**N(%)**	**N(%)**	**N(%)**	**T1-T2 †**	**T1-T3 †**	**T2-T3 †**
Satisfy general curiosity<br>Median		19(100%)<br>Strongly agree	19(100%)<br>Strongly agree	17(89.5%)<br>Strongly agree	z=-0.45, p=0.66, r=-0.10	z=-1.29, p=0.20, r=0.30	z=-1.89, p=0.059, r=0.43
Understand what a patient may learn/experience<br>Median		18(94.7%)<br>Strongly agree	18(94.7%)<br>Agree	16(84.2%)<br>Agree	z=-1.51, p=0.13, r=0.35	z=-1.94, p=0.052, r=0.45	z=-0.82, p=0.41, r=0.19
Help understand principles of human genetics<br>Median		18(94.7%)<br>Strongly agree	15(78.9%)<br>Agree	17(89.5%)<br>Agree	z=-1.89, p=0.059, r=0.43	**z=-2.12, p=0.034, r=0.49**	z=-0.33, p=0.74, r=0.08
See if a specific disease runs in the family or is in DNA<br>Median		15(78.9%)<br>Agree	17(89.5%)<br>Agree	16(84.2%)<br>Agree	z=-1.73, p=0.083, r=0.40	z=-0.75, p=0.45, r=0.17	z=-1.03, p=0.31, r=0.24
Inform family members about health risks<br>Median		11(57.9%)<br>Agree	13(68.4%)<br>Agree	9(47.4%)<br>Neither	z=-1.39, p=0.16, r=0.32	z=-0.30, p=0.76, r=0.07	**z=-2.13, p=0.033, r=0.49**
Learn about genetic makeup without going through a physician<br>Median		9(47.4%)<br>Neither	13(68.4%)<br>Agree	12(63.2%)<br>Agree	z=-1.81, p=0.071, r=0.42	z=-1.52, p=0.13, r=0.35	z=-0.83, p=0.41, r=0.19
**Reasons *against* using own genome (agree / strongly agree)**		**N(%)**	**N(%)**	**N(%)**	**T1-T2 †**	**T1-T3 †**	**T2-T3 †**
Concern about privacy/risks to privacy<br>Median		10(52.6%)<br>Agree	6(31.6%)<br>Disagree	5(26.3%)<br>Neither	**z=-2.39, p=0.017, r=0.55**	z=-2.22, p=0.026, r=0.51	z=-0.79, p=0.43, r=0.18
Results are not predictive<br>Median		10(52.6%)<br>Agree	11(57.9%)<br>Agree	10(52.6%)<br>Agree	z=-0.59, p=0.56, r=0.14	z=-0.27, p=0.79, r=0.06	z=-1.35, p=0.18, r=0.31
Unwanted information<br>Median		6(31.6%)<br>Disagree	6(31.6%)<br>Neither	8(42.1%)<br>Neither	z=-0.11, p=0.91, r=0.03	z=-0.26, p=0.0.80, r=0.06	z=-0.88, p=0.38, r=0.20
Results are not reliable<br>Median		3(15.8%)<br>Disagree	6(31.6%)<br>Neither	4(21.1%)<br>Disagree	z=-1.35, p=0.18, r=-0.31	z=-0.54, p=0.59, r=0.12	z=-1.77, p=0.078, r=0.41
Results are not accurate<br>Median		3(15.8%)<br>Disagree	5(26.3%)<br>Neither	3(15.8%)<br>Disagree	z=-1.43, p=0.15, r=0.33	z=-0.80, p=0.43, r=0.18	z=-1.36, p=0.17, r=0.31
Information will not be medically useful/will not change medical decisions<br>Median		1(5.3%)<br>Disagree	5(26.3%)<br>Neither	3(15.8%)<br>Disagree	z=-0.21, p=0.84, r=0.05	z=-0.56, p=0.58, r=0.13	z=-0.86, p=0.39, r=0.20
Costs too much<br>Median		2(10.5%)<br>Neither	2(10.5%)<br>Neither	2(10.5%)<br>Disagree	z=-1.25, p=0.21, r=0.29	z=-2.06, p=0.039, r=0.47	z==1.49, p=0.14, r=0.34

Information will not help learn human genetics<br>Median	0(0%)<br>Disagree	0(0%)<br>Disagree	0(0%)<br>Disagree	z=-0.07, p=0.94, r=0.02	z=0.00, p=0.99, r=0.00	z=-0.45, p=0.66, r=0.10
**Perceived benefits and concerns about educational WGS (agree / strongly agree)**	**T1 N(%)**	**T2 N(%)**	**T3 N(%)**	**T1-T2 †**	**T1-T3 †**	**T2-T3 †**
I would see this as an opportunity to get a service that I would not ordinarily get if I had to pay full price.<br>Median	16(84.2%)<br>Agree	17(89.5%)<br>Agree	17(89.5%)<br>Agree	z=-0.37, p=0.71, r=0.08	z=-0.35, p=0.73, r=0.08	z=0.00, p=0.99, r=
I would be concerned that I might get some results that would be disturbing.<br>Median	15(78.9%)<br>Agree	13(68.4%)<br>Agree	14(73.7%)<br>Agree	z=-0.63, p=0.53, r=0.14	z=0.00, p=0.99, r=0.00	z=-1.00, p=0.32, r=0.23
I would see this as an opportunity to get information that would help me improve my health.<br>Median	13(68.4%)<br>Agree	17(89.5%)<br>Agree	13(68.4%)<br>Agree	z=-1.51, p=0.13, r=0.35	z=0.00, p=0.99, r=0.00	z=-1.51, p=0.13, r=0.35
My own results would help me understand genetics concepts better than someone else's results.<br>Median	8(42.1%)<br>Neither	15(78.9%)<br>Agree	12(63.2%)<br>Agree	**z=-2.12, p=0.034, r=0.49**	z=-1.78, p=0.075, r=0.41	z=-1.17, p=0.24, r=0.27
I would be concerned that people would find out genetic or health information about me.<br>Median	6(31.6%)<br>Neither	2(10.5%)<br>Disagree	4(21.1%)<br>Disagree	**z=-2.49, p=0.013, r=0.57**	z=-2.23, p=0.026, r=	z=-1.31, p=0.19, r=0.30
I would only take up the offer of testing if I could get genetic counseling after I got my results back in.<br>Median	4(21.1%)<br>Disagree	5(26.3%)<br>Disagree	6(31.6%)<br>Neither	z=-0.54, p=0.59, r=0.12	z=0.00, p=0.99, r=0.00	z=-0.58, p=0.56, r=0.13
I feel that I would be at a disadvantage to my classmates if I did not undergo the testing.<br>Median	2(10.5%)<br>Disagree	3(15.8%)<br>Disagree	2(10.5%)<br>Disagree	z=-0.11, p=0.92, r=0.03	z=-0.58, p=0.56, r=0.13	z=-0.57, p=0.57, r=0.13
I would be concerned that my professors would know who took up the offer of testing and who did not.<br>Median	2(10.5%)<br>Disagree	1(5.3%)<br>Disagree	2(10.5%)<br>Disagree	z=-1.27, p=0.21, r=0.29	z=-1.41, p=0.16, r=0.32	z=0.00, p=0.99, r=0.00
I would be concerned that my classmates would know who took up the offer of testing and who did not.<br>Median	2(10.5%)<br>Disagree	2(10.5%)<br>Disagree	5(26.3%)<br>Disagree	z=-0.35, p=0.73, r=0.08	z=-0.70, p=0.48, r=0.16	z=-1.30, p=0.19, r=0.30
I would only take up the offer of testing if I could get genetic counseling before I sent my sample in.<br>Median	2(10.5%)<br>Disagree	2(10.5%)<br>Disagree	3(15.8%)<br>Disagree	z=-1.00, p=0.32, r=0.23	z=-1.51, p=0.13, r=0.35	z=-2.31, p=0.021, r=0.53

† Differences assessed using Wilcoxon's signed-rank test (Wilcoxon, 1945: the nonparametric equivalent of the dependent or paired samples t-test) in each case to test the null hypothesis that the responses between the time points were identical

[a] Note that significance tests used all available data, i.e. all 5 response options, instead of the dichotomized responses displayed in the Table

[b] One person gave an "Other" response, which was that at T3, one student stated "I just think its very interesting to further expand the knowledge of self"

Supplemental Table 3. Students' attitudes towards personal whole genome sequencing in general

Attitudes towards WGS in general	T1 N(%)	T2 N(%)	T3 N(%)	T1-T2 †	T1-T3 †	T2-T3 †
If I underwent whole genome sequencing, I would ask a physician for help in interpreting the results. Agree/strongly agree:<br>Median	9(47.4%)<br>Neither	8(42.1%)<br>Neither	5(26.3%)<br>Disagree	z=-0.92, p=0.36, r=0.21	z=-2.00, p=0.046, r=0.46	z=-1.64, p=0.10, r=0.38
How likely is it that knowing the results from whole genome sequencing for yourself would lead to any changes in your behavior? Quite likely/very likely:<br>Median	8(42.1%)<br>Not sure	5(26.3%)<br>Not sure	7(36.8%)<br>Not sure	z=-1.12, p=0.27, r=0.26	z=0.00, p=0.99, r=0.00	**z=-2.24, p=0.025, r=0.51**
Results of whole genome sequencing would influence my future health care decisions. Agree/strongly agree:<br>Median	13(68.4%)<br>Agree	11(57.9%)<br>Agree	13(68.4%)<br>Agree	z=-0.45, p=0.66, r=0.10	z=0.00, p=0.99, r=0.00	z=-0.58, p=0.56, r=0.13
How useful do you think the results from whole genome sequencing information will be to patients themselves? Useful/very useful:<br>Median	10(52.6%)<br>Useful	7(36.8%)<br>Not sure	9(47.4%)<br>Not sure	z=-0.63, p=0.53, r=0.14	z=0.00, p=0.99, r=0.00	z=-0.63, p=0.53, r=0.14
Most people can accurately interpret whole genome sequencing results. Agree / strongly agree:<br>Median	0(0%)<br>Strongly disagree	0(0%)<br>Strongly disagree	0(0%)<br>Strongly disagree	z=-0.45, p=0.66, r=0.10	z=-0.38, p=0.71, r=0.09	z=0.00, p=0.99, r=0.00
How useful do you think the results from whole genome sequencing will be to a physician? Useful/very useful:<br>Median	9(47.4%)<br>Not sure	12(63.2%)<br>Useful	10(52.6%)<br>Agree	z=-0.05, p=0.96, r=0.01	z=-0.14, p=0.89, r=0.03	z=-0.14, p=0.89, r=0.03
Physicians have enough knowledge to help individuals interpret results of whole genome sequencing. Agree/strongly agree:<br>Median	1(5.3%)<br>Disagree	1(5.3%)<br>Disagree	0(0%)<br>Disagree	z=-1.10, p=0.27, r=0.25	z=-1.27, p=0.21, r=0.29	z=-0.45, p=0.66, r=0.11
Physicians have a professional responsibility to help individuals understand the results they receive from whole genome sequencing, even if the physician has not ordered the test. Agree/strongly agree:<br>Median	11(57.9%)<br>Agree	8(42.1%)<br>Neither	8(42.1%)<br>Neither	z=-0.87, p=0.39, r=0.20	z=2.06, p=0.040, r=0.47	**z=-2.11, p=0.035, r=0.48**
I understand the risks and benefits of using getting personal whole genome sequencing done. Agree/strongly agree:<br>Median	11(57.9%)<br>Agree	17(89.5%)<br>Agree	18(94.7%)<br>Agree	**z=-2.71, p=0.007, r=0.62**	z=-2.67, p=0.008, r=0.61	z=-0.82, p=0.41, r=0.19
I know enough about genetics to understand the whole genome sequencing results. Agree/strongly agree:<br>Median	6(31.6%)<br>Disagree	8(42.1%)<br>Neither	7(36.8%)<br>Neither	z=-1.64, p=0.10, r=0.38	z=2.23, p=0.026, r=0.51	z=-1.67, p=0.096, r=0.38
Whole genome sequencing is useful for patients. Agree/strongly agree:<br>Median	14(73.7%)<br>Agree	13(68.4%)<br>Agree	14(73.7%)<br>Agree	z=-1.00, p=0.32, r=0.23	z=-0.45, p=0.66, r=0.10	z=-0.82, p=0.41, r=0.19

† Differences assessed using Wilcoxon's signed-rank test (Wilcoxon, 1945: the nonparametric equivalent of the dependent or paired samples t-test). Significance tests used all available data, i.e. all 5 response options, instead of the dichotomized responses displayed. Effects are reported at the 0.05 level of significance.

Supplemental Table 4. Students' knowledge about personal genomic results based on three clinical scenarios

Knowledge question	T1 N(%)	T2 N(%)	T3[1] N(%)	T1-T2 †	T1-T3 †	T2-T3 †
**SCENARIO 1: BREAST CANCER**						
**1.A. Interpretation of results**						
Patient has higher risk than average	8(42.1%)	7(36.8%)	8(42.1%)	z=0.58, p=0.56, r=0.13	z=-0.58, p=0.56, r=0.13	z=-1.00, p=0.32, r=0.23
A different genetic test should be ordered	5(26.3%)	8(42.1%)	9(47.4%)	z=-1.73, p=0.083, r=0.40	z=-2.00, p=0.046, r=0.46	z=-1.00, p=0.32, r=0.23
**1.B. What issues impacted your understanding of the case?**						
Family history	12(63.2%)	13(68.4%)	15(78.9%)	z=-0.38, p=0.71, r=0.09	z=-2.00, p=0.046, r=0.46	z=-1.34, p=0.18, r=0.31
**1.C. How would you counsel the patient?**						
Should have clinical screening for breast cancer	15(78.9%)	15(78.9%)	16(88.9%)	z=0.00, p=0.99, r=0.00	z=-0.58, p=0.56, r=0.13	z=-1.00, p=0.32, r=0.23
**SCENARIO 2: HEMOCHROMATOSIS**						
**2.A. Interpretation of results**						
Patient is a carrier of hemochromatosis and may develop it*	2(10.5%)	4(21.1%)	2(10.5%)	z=-1.00, p=0.32, r=0.23	z=0.00, p=0.99, r=0.00	z=-1.00, p=0.32, r=0.23
Patient has no risk for hemochromatosis*	15(78.9%)	11(57.9%)	13(68.4%)	z=-1.41, p=0.16, r=0.32	z=-0.82, p=0.41, r=0.19	z=-0.82, p=0.41, r=0.19
**2.B. What issues impacted your understanding of the case?**						
Mode of inheritance	15(78.9%)	16(84.2%)	16(84.2%)	z=-0.45, p=0.66, r=0.10	z=-1.00, p=0.32, r=0.23	z=-0.58, p=0.56, r=0.13
Penetrance of the condition	5(26.3%)	5(26.3%)	5(26.3%)	z=0.00, p=0.99, r=0.00	z=-0.45, p=0.66, r=0.10	z=-0.33, p=0.74, r=0.08
**2.C. How would you counsel the patient?**						
Not at increased risk but should let family know they are a carrier and others may be at risk	10(52.6%)	14(73.7%)	15(78.9%)	z=-1.41, p=0.16, r=0.32	z=-1.89, p=0.059, r=0.43	z=-0.38, p=0.71, r=0.09
**SCENARIO 3: MACULAR DEGENERATION**						
**1.A. Interpretation of results**						
Patient has higher risk than average	8(42.1%)	9(47.4%)	7(36.8%)	z=-0.45, p=0.66, r=0.10	z=0.00, p=1.00, r=0.00	z=-0.45, p=0.66, r=0.10
**1.B. What issues impacted your understanding of the case?**						
Test results and interpretation by the company	8(42.1%)	6(31.6%)	9(47.4%)	z=-0.82, p=0.41, r=0.19	z=-0.38, p=0.71, r=0.09	z=-1.13, p=0.26, r=0.26

None of the above	5(26.3%)	5(26.3%)	4(21.1%)	z=0.00, p=0.99, r=0.00	z=-0.58, p=0.56, r=0.13	z=-0.45, p=0.66, r=0.10
**1.C. How would you counsel the patient?**						
Should have clinical screening for macular degeneration	8(42.1%)	11(57.9%)	9(47.4%)	z=-1.73, p=0.083, r=0.40	z=-0.38, p=0.71, r=0.09	z=-0.82, p=0.41, r=0.19

† Differences assessed using Wilcoxon's signed-rank test.
Note: Response options shown are those deemed to be the 'best' by Ormond et al (2011). Response options that were deemed to be 'incorrect' are not displayed here.
*Because the item viewed as 'best' by Ormond et al (2011) was not included in the present questionnaire, we allowed these 2 items to be coded as 'best'.
[1] One student did not complete this part of the questionnaire at T3.

Supplemental Table 5. Students' responses to the open-ended questions comments sections of the questionnaires

Pre-introductory course comments (T1)	Post-introductory course comments (T2)	Pre-advanced course comments (T3)
[1] I am honored to be part of this course and to have the opportunity to look at my own genome with all the risks and benefits associated. I also think that as we evolve into the personal medicine era it will allow me to better speak and understand the science. Lastly I think that a better view on the genomic level will prove my understanding of the [redacted] which my research focuses on. Thanks for letting me be a part.	[1] Can't wait to actually work with my own genome!!! The introductory course was amazing. I am aware to the possibility of getting a lot of non informative results but still some. In regards to changing my lifestyle I think it is very well adopted to my needs so I am more excited to learn about other variants, more of the late onset phenotype. Thank you.	I am so happy to be here!!!!!
[2] Main concerns: privacy, unavailability of treatments or cure for many genetic diseases.	[2] Just would like to know the experience that other person went through	
	[5] Need good consent process so patient can understand what is likely options for them	
		[6] Education on interpretation is great, but ultimately much more didactic training on basic analyses are needed for anyone in this class to be able to actually analyze sequence data independently outside of this class, also, for the knowledge questions, you should probably have an "i don't know" option for all questions, not just some.
	[7] Specific concerns about issues such as privacy, ownership of the genomic information, publication or other forms of access to genomic information by third parties were not addressed during the summer course	
	[8] The course instructors were excellent and were able to tailor the classes to students with very different backgrounds in genetics and computational skills. Thank you for a great course.	
[9] Will genetic counseling or referral be available to students in this course prior to, or after whole genome sequencing? Are physician geneticists available, if needed for counseling or further testing?	[9] I am very interested and I hope to get the opp. to sequence my own genome	
[10] I would be and am very tempted very interested in sequencing my own genome, but my fear of detecting	[10] The fact that many variants are so difficult to interpret makes the decision very difficult (whether to sequence my own genome or not).	

susceptibilities/predispositions and therefore higher roles of developing certain diseases will probably keep me from doing so, I am curious to see if I will change my mind after taking this course.	Overall, even though I obtained better insight into the process of genome/exome interpretation, I am still very undecided and the fear of finding variants that carry a sig. disease risk is by no means decreased.	
[11] I think another potential benefit of genome sequencing is to help alleviate reproductive anxieties in the future. Working in genetics shows us a lot of what can go wrong and that residual risks after negative carrier screening is for common mutations, don't yield a 0% chance of having an affected offspring. This more expansive information can help in making more informed reproductive choices.		
		[12] I think the course needs a better instructor/student ratio. more instructors please! also, if we get more instructors we can work in small groups so less time lost with instructors walking around. more longitudinal focus on student development
[13] What is reportable? How to consent for different types of disorders? Do we group them, mitochondrial? hearing loss? treatable? adult onset? And then how do we classify disorders into these groups? Will there be an info. letter to provide to closely related family members?		
[14] I am concerned if the info. from the sequencing will affect life insurance. Also will all of the course directors know the results of your personal sequencing. Who will be doing the counseling and have availability to the results?	[14] I think it would be helpful to have a dictionary of commands for inputting filters etc. I think it would also be helpful to have list of genes of common conditions or a way to search for specific types of SNPs like "cancer". Also an intro to the class about what is and is not detectable by WGS. [redacted] were great!	[14] These questions go much further into clinical genetics and principle that have been ignored in class. we need to focus more on result interpretation than coding
[16] The course sounds very interesting, I am not sure if it will be more educational because we analyze our own genomes. This might be disturbing.	[16] That was a great course, looking fw to the fall. It would be helpful to include more case studies like the ones in the survey to help with interpretation of results.	
[17] Include on Blackboard some more multimedia resources, for instance, PBS documentary with Francis Collins, NY Times recent piece on personalized medicine.	[17] Compile some patient vignettes about how they feel about WGS	
	[19] I feel that WGS of healthy individuals is recreational. I don't believe that GWAS SNPS or odds ratios are useful. Useful info. from WGS	

	would be disease gene status (carrier or affected). I would not want to know untreatable adult-onset disorders. I have no significant family hx that makes me curious about being affected with a genetic disorder. I can order expanded carrier screening through my doctor and it is covered by insurance. So, most clinically significant carrier statuses would be best found out that way if I chose. I believe that a "mock" genome would be just as useful to my learning experience in this course.	

[N] denotes subject ID
