# Supplementary material for: Informed decision-making among students analyzing their personal genomes on a whole genome sequencing course: a longitudinal cohort study
Source: Genome Med. 2013 Dec 30;5(12):113. doi: 10.1186/gm518 (PMC3971344; doi:10.1186/gm518)
Supplement: Additional file 6 — Syllabus. [file gm518-S6.docx]

**SYLLABUS**

**Part 1 (introductory course): Introduction to Human Genome Sequencing**

**July 23: Class Introduction and ELSI**

Present ethical, legal and social implications (ELSI) of genetic testing, both focused genotyping and whole genomes sequencing, as it relates to both patients and their families, and potential children. Provide context for the decisions facing patients, researchers and clinicians.

**July 24: Introduction to High-Performance Computing**

Introduce the computing, software and data resources to be used for the class, including how to obtain; access; and use compute clusters securely and efficiently.

**July 25: Introduction to 2nd Generation Sequencing**

Tour the Mount Sinai sequencing facility and present the entire sequencing workflow. Discuss the importance of CLIA and other certifications. Present limitations imposed by the sequencing technology itself. Goal is to fully understand the limitations and sources of error in the sequencing technology.

**July 26: Short-read Mapping and Calibration: FASTQs to BAMs**

Introduce front-end of data pipeline for 2nd generation DNA sequencing technology including (re)alignment and recalibration. Present commonly used read mapping algorithms and tools, and the strengths and limitations thereof. Run mapping tools with genome data.

**July 27: Variant Calling: BAMs to VCF**

Introduce back-end of data pipeline including variant calling for SNVs and indels and variant filtering. Call variants in genomic data. Focus on various sources of error in filtering, mapping and variant detection with practical experimentation with different parameter settings for variant detection tools.

**July 30: Review Calling Results; Introduction to Annotation**

Review variant calling results with a focus on important quality metrics. Introduce tools and data resources used in annotating and interpreting a personal genome

**July 31: Pharmacogenomics**

Explore variants with known pharmacogenetic effects in context of lab genomes. Build a pharmacogenomics profile for the lab genomes.

**August 1: Disease Risk**

Analyze found SNVs associated with disease risk in the context of public databases, literature and other resources. Introduce interpretation of SNV findings (causal vs. not, multi-allelic models) and the limitations thereof.

**August 2: Mendelian Disease**

Build hypotheses of the nature of disease causing variant and translate those hypotheses into queries against the called variants. Introduce how variants could be ranked for likely pathogenic effect.

**August 3: Ancestry**

Introduce how ancestry and historical human relationships can be inferred from genetic information. Infer the ancestry of the laboratory genomes.

**August 6: Communicating and Responding to Genetic Results**

Introduce how genetic testing results are communicated to patients, with particular focus on whole genome sequencing. Review current understanding of how patients respond to genetic testing results emotionally and behaviorally.

**August 7-8: Capstone Exercise**

Students will select a small number of variants from the laboratory genomes on which to perform deep analysis of clinical impact. These “deep dives” will include both discussion of whether a variant is significant and should be reported and the “ clinical reporting” of their findings in a role-playing exercise. Issues related to the interpretation of the significance of genomic variants and how to communicate these findings will be explored in the context of potential clinical scenarios.

**Part 2 (advanced course): Practical Analysis of a Personal Genome**

**Course meets Wednesday 4-6 PM and Friday 3-5 PM in Levy Library (11-41)

1. 9/12: Questionnaire, Informed Consent Refresher

2. 9/14: Bogus Results in Human Genetics

3. 9/19: Report-ability, disease classification, disease to gene to coordinates

4. 9/21: Family history applied to risk, phasing, etc.

5. 9/28: Gene Inclusion/Exclusion

6. 10/3: Complex Traits, Risk Models, GWAS

7. 10/5: Curation report

8. 10/10: VUS: Structure of disease, human genetic variation

9. 10/12: VUS: Annotation … conservation, biochemical, etc.

10. 10/17: VUS: Putting it all together

11. 10/19: Tour of Sequencing Facility

12. 10/24: Mechanics of working with my sequence

13. 10/26: Alignment/Mapping

14. 10/31: Variant Calling/Error Modeling/QC

15. 11/2: My genome: Ancestry

16. 11/7: My genome: Carrier Status, PGx, Complex Traits

17. 11/9: My genome: Carrier Status, PGx, Complex Traits

18. 11/14: My genome: Carrier Status, PGx, Complex Traits

18. 11/16: My genome: VUS

19. 11/28: T2D, Group project setup and working time

20. 11/30: Physical traits/burden testing

21. 12/5: X-linked, regulatory variants

22. 12/7: Group presentations, discussion

23. 12/12: Questionnaire**
