# Supplementary material for: Informed decision-making among students analyzing their personal genomes on a whole genome sequencing course: a longitudinal cohort study
Source: Genome Med. 2013 Dec 30;5(12):113. doi: 10.1186/gm518 (PMC3971344; doi:10.1186/gm518)
Supplement: Additional file 7 — Participant information sheet for the research component including the T1 and T2 questionnaires. [file gm518-S7.pdf]

Mount Sinai School of Medicine IRB Approved Program for the Protection of Human Subjects

TITLE OF RESEARCH STUDY:

Students' Attitudes Towards the Use of Personal Genome Data in the Classroom

PRINCIPAL INVESTIGATOR (HEAD RESEARCHER) NAME AND CONTACT INFORMATION:

Name: Dr. Saskia Sanderson

Physical Address: 1425 Madison Avenue, 3rd Floor

Mailing Address: 1 Gustave L Levy Place Box 1498, NY, NY 10029

Phone: 212-659-8520

WHAT IS A RESEARCH STUDY?

A research study is when scientists try to answer a question about something that we don't know enough about. Participating may not help you or others.

People volunteer to be in a research study. The decision about whether or not to take part is totally up to you. You can also agree to take part now and later change your mind. Whatever you decide is okay. Your decision whether or not to participate will not affect your grades in the "Introduction to Human Genome Sequencing" course or any other aspect of your education at Mount Sinai School of Medicine. You can continue to take the "Introduction to Human Genome Sequencing" course even if you choose not to participate in the research study.

Someone will explain this research study to you. Feel free to ask all the questions you want before you decide. Any new information that develops during this research study which might make you change your mind about participating will be given to you promptly.

PURPOSE OF THIS RESEARCH STUDY:

The purpose of this study is to learn about students' attitudes towards having the option of analyzing their own genomes as part of the class process when learning about whole genome sequencing. Whole genome sequencing means that scientists analyze your DNA (genetic material inherited from your parents that determines your traits) to obtain a complete and detailed listing of all of your genetic material (your "genome"). Your genome is what makes you unique, as it contains all of the information that determines your physical characteristics, your risks of inheriting certain diseases, and your metabolism. Our goal in this research study is to learn more about how students feel about analyzing their own genome data in the classroom.

You may qualify for participation in this study because you are enrolled on the "Introduction to Human Genome Sequencing" course at Mount Sinai School of Medicine.

Funds for conducting this research are provided by the Mount Sinai School of Medicine's Institute for Genomics and Multiscale Biology.

LENGTH OF TIME AND NUMBER OF PEOPLE EXPECTED TO PARTICIPATE

Your participation in this research study is expected to last approximately 1 hour in two separate classes.

Mount Sinai School of Medicine
I R B Approved
Program for the Protection of Human Subjects

The number of people expected to take part in this research study at this site is 20 people per year.

DESCRIPTION OF WHAT'S INVOLVED:

If you agree to participate in this research study, the following information describes what may be involved. Please note that all study procedures are being done for research purposes only.

Precourse questionnaire (15-30 mins)
Before you start your first class on the "Introduction to Human Genome Sequencing" course, you will be asked to answer some questions to assess your interest in analyzing your own genome as part of a future advanced course on whole genome sequencing, and your perceived benefits and concerns about doing this. These questions will be administered as a paper questionnaire. You will then commence with your first class and the rest of the course.

Postcourse questionnaire (15-30 mins)

At the end of your last class on the "Introduction to Human Genome Sequencing" course, you will be asked to complete an identical questionnaire to the one you completed in the first class. As with the first questionnaire, the point of this questionnaire is to assess your attitudes towards analyzing your own genome data as part of an advanced whole genome sequencing course. We are also interested in whether your attitudes have changed over time.

Your questionnaires will be identified only by a study number; your name and other information that could identify you will not be on the questionnaires. The study number will be "linked" to your name in a secure database which will not be accessible by any of the course instructors. This is to ensure that the instructors will not know if you are participating in the study, or what your answers to the questionnaires are.

YOUR RESPONSIBILITIES IF YOU TAKE PART IN THIS RESEARCH:

If you decide to take part in this research study you will be responsible for the following things: completing the precourse questionnaire and completing the postcourse questionnaire.

COSTS OR PAYMENTS THAT MAY RESULT FROM PARTICIPATION:

If you agree to take part in this research study, you will not be paid for participation.

POSSIBLE BENEFITS:

It is important to know that you may not get any benefit from taking part in this research study. Others may not benefit either.

REASONABLY FORESEEABLE RISKS AND DISCOMFORTS:

Risks related to privacy:
There always exists the potential for loss of private information; however, there are procedures in place to minimize this risk.

Mount Sinai School of Medicine IRB Approved Program for the Protection of Human Subjects

OTHER POSSIBLE OPTIONS TO CONSIDER:

You may decide not to take part in this research study without any penalty. The choice is totally up to you.

IN CASE OF INJURY DURING THIS RESEARCH STUDY:

If you believe that you have suffered an injury related to this research as a participant in this study, you should contact the Principal Investigator.

ENDING PARTICIPATION IN THE RESEARCH STUDY:

You may stop taking part in this research study at any time without any penalty. This will not affect your participation, grade or any other aspect of your involvement on the “Introduction to Human Genome Sequencing” course, or any other aspect of your education at Mount Sinai School of Medicine.

If you decide to stop being in the research study, please contact the Principal Investigator or the research staff.

You may also withdraw your permission for the use and disclosure of any of your protected information for research, but you must do so in writing to the Principal Investigator at the address on the first page. Even if you withdraw your permission, the Principal Investigator for the research study may still use the information that was already collected if that information is necessary to complete the research study.

Withdrawal without your consent: The study team or the institution may stop your involvement in this research study at any time without your consent. This may be because the research study is being stopped, the instructions of the study team have not been followed, the investigator believes it is in your best interest, or for any other reason.

CONTACT PERSON(S):

If you have any questions, concerns, or complaints at any time about this research, or you think the research has hurt you, please contact Dr. Sanderson at telephone number 212-659-8520.

This research has been reviewed and approved by an Institutional Review Board. You may reach a representative of the Program for Protection of Human Subjects at Mount Sinai School of Medicine at telephone number (212) 824-8200 during standard work hours for any of the following reasons:

- Your questions, concerns, or complaints are not being answered by the research team.
- You cannot reach the research team.
- You are not comfortable talking to the research team.
- You have questions about your rights as a research subject.
- You want to get information or provide input about this research.

DISCLOSURE OF FINANCIAL INTERESTS:

None.
