# Supplementary material for: Informed decision-making among students analyzing their personal genomes on a whole genome sequencing course: a longitudinal cohort study
Source: Genome Med. 2013 Dec 30;5(12):113. doi: 10.1186/gm518 (PMC3971344; doi:10.1186/gm518)
Supplement: Additional file 8 — Participant information sheet for the research component including the T3 questionnaire. [file gm518-S8.doc]

Title: Personal Genome Analysis in the Classroom Setting

Researcher: Saskia Sanderson, PhD

1425 Madison Ave, 3rd floor, Room 3-72

212-659-8520

The main purpose of this study is to explore the pros and cons of offering students the option of analyzing and interpreting their own, personal genome data as part of a whole genome sequencing (WGS) course. Our first goal is to explore what choices students make when they have been offered the option of analyzing their own genome data, i.e. whether students choose to analyze and interpret their own genomes as part of the course, or instead prefer to analyze and interpret an anonymous publicly available genome. Our second goal is to explore the potential benefits (e.g. improved engagement with the course content, improved understanding of the implications of WGS-based results for patients), and the potential harms (e.g. finding the personal information obtained distressing) of the course.

You may qualify for participation in this study because you took the Mount Sinai School of Medicine (MSSM) “Introduction to Human Genome Sequencing” course, and are enrolled on the MSSM “Practical Analysis of Your Personal Genome” course.

Funds for conducting this research are provided by the MSSM Institute for Genomics and Multiscale Biology.

Being in a research study is completely voluntary. You can choose not to be in this research study. You can also say yes now, and change your mind later. Your decision whether or not to participate will not affect your grades in the “Practical Analysis of Your Personal Genome” course or any other aspect of your education at Mount Sinai School of Medicine. You can continue to take the “Practical Analysis of Your Personal Genome” course even if you choose not to participate in the research study.

If you agree to take part in this research, you will be asked to complete a precourse questionnaire and a postcourse questionnaire. Your participation in this study will take about 15-30 minutes on the first and last day of class. We expect that 20 people will take part in this research study.

The possible risks to you in taking part in this research are that you may feel uncomfortable answering some of the questions, and the risk of potential loss of private information.

You can choose not to answer any question you do not wish to answer. You can also choose to stop taking the survey at any time.

To protect your identity as a research subject, *Your questionnaires will be identified only by a study number; your name and other information that could identify you will not be on the questionnaires. The study number will be “linked” to your name in a secure database which will not be accessible by any of the course instructors. This is to ensure that the instructors will not know if you are participating in the study, or what your answers to the questionnaires are.*

There are no direct benefits to you for being in this research study. The possible benefits of this research are in helping us understand the pros and cons of providing students with the option of analyzing their own personal genome data which may help students who might choose to enroll in whole genome sequencing courses of this type in the future.

If you have any questions about this research, or feel that you have been injured from taking part in the research, please contact the PI, Dr. Saskia Sanderson at 212-659-8520. If you have any questions about your rights as a study subject, please contact the Program for the Protection of Human Subjects at 212-824-8200 or [IRB@mssm.edu](mailto:IRB@mssm.edu).
