# Supplementary material for: Informed decision-making among students analyzing their personal genomes on a whole genome sequencing course: a longitudinal cohort study
Source: Genome Med. 2013 Dec 30;5(12):113. doi: 10.1186/gm518 (PMC3971344; doi:10.1186/gm518)
Supplement: Additional file 9 — Information sheet for the personal whole genome sequencing component of the ‘Practical analysis of your personal genome’ course. [file gm518-S9.doc]

| TITLE OF COURSE: |
| --- |

**Practical Analysis of Your Personal Genome Course:**

**Personal Whole Genome Sequencing Component**

| PURPOSE OF WHOLE GENOME SEQUENCING: |
| --- |

You are being offered personal whole genome sequencing (WGS) as part of the educational course, Practical Analysis of Your Personal Genome. The main purpose of the course is to educate you, the student, about WGS.

A concurrent study is being conducted to explore the pros and cons of offering students the option of analyzing and interpreting their own, personal genome data as part of the WGS course. Our first goal in that study is to explore what choices students make when they have been offered the option of analyzing their own genome data, i.e. whether students choose to analyze and interpret their own genomes as part of the course, or instead prefer to analyze and interpret an anonymous publicly available genome. Our second goal in that study is to explore the potential benefits (e.g. improved engagement with the course content, improved understanding of the implications of WGS-based results for patients), and the potential harms (e.g. finding the personal information obtained distressing) of the course.

**You may qualify to have your genome sequenced** because you took the Mount Sinai School of Medicine (MSSM) “Introduction to Human Genome Sequencing” course, and are enrolled on the MSSM “Practical Analysis of Your Personal Genome” course.

Funds for conducting this sequencing are provided by the MSSM Institute for Genomics and Multiscale Biology.

Note that WGS is currently not a clinically approved test in New York State and is being done for educational purposes only.

| LENGTH OF TIME AND NUMBER OF PEOPLE EXPECTED TO PARTICIPATE IN WGS FOR THIS COURSE |
| --- |

This is the information sheet for the personal WGS Component of the WGS course.

Participation in the WGS Component of the course is expected to last the duration of the course (including blood draw for sequencing, return of sequence data to the students, the students analyzing their own genome sequence data for educational purposes). Thus the total duration of your participation in the WGS Component of the course is expected to be about 3.5 months.

The number of people expected to take part in this course at this site is 15-20 people.

| DESCRIPTION OF WHAT’S INVOLVED: |
| --- |

If you agree to participate in the personal sequencing component of this course, the following information describes what may be involved. Please note that all procedures (counseling sessions, whole genome sequencing, etc.) are being done for educational purposes only and will not be part of your permanent medical record.

**Optional Pre-WGS Genetic Counseling**

If you wish to discuss whether or not to obtain your own personal WGS data with a healthcare professional, you can contact Claire Davis at MSSM (212-241-6149) or Jill Goldman at Columbia University (212-305-7382) to make an appointment. This will be made available to you free of charge.

**WGS Information and Blood Draw Session**

The “Introduction to Human Genome Sequencing” course provided detailed background into the types of information that can be learned from whole genome sequences and the limitations thereof. Here, we would like to remind you of the following:

1. It is expected that you will find a large number of unique variants in their DNA that may be difficult to interpret at the present time;
2. Your personal raw data from WGS is being provided to you for informational, educational and research purposes only, and are not intended to substitute for a medical diagnosis;
3. The amount of information that will be generated from your WGS will be vast and may be of unclear significance to your health;
4. Our knowledge about the meaning of variations in the genome is growing rapidly but is largely incomplete;
5. Potentially important variants that may be important to your health may be missed;
6. Some variants may be identified that are likely to be of clinical significance;
7. Some of this information can be disturbing;
8. It is very important for you to carefully think about whether you wish to analyze your own genome data through which many different types of personal information may arise.

We would like to remind you that the types of variants that you might find in your DNA include (but are not limited to):

- Variants related to ancestry;
- Variants in genes that affect how your body uses certain medications (pharmacogenomics);
- Variants in genes that might increase or decrease your risk of developing conditions like heart disease and obesity;
- Variants in genes that might increase or decrease your risk of developing certain types of cancers;
- Variants in genes that might increase or decrease your risk of developing psychiatric disorders, such as schizophrenia, and neurologic disorders, such as Alzheimer’s disease;
- Variants that indicate that you are a carrier for a disease gene (i.e. that you have one “disease” copy of a gene, and one healthy copy of the same gene, that while a carrier does not have the disease, there is a chance that family members might either have the disease or also be carriers, and that if you do find that you are a carrier of a disease gene, you may wish to review whether any family members might be at risk, and if so, how to approach them to discuss testing);
- Variants that indicate that you have a treatable disease that you were not aware of (if you find that you have a disease that is treatable, you may wish to be referred for appropriate confirmatory testing and treatment);
- Variants that indicate that you are likely to develop a serious, potentially fatal disease, for which there is no treatment or cure.

It is also possible that no information of any clinical significance will be revealed.

Please note that this is an academic class and that the sequencing is not being performed under a clinical protocol. No results can or should be used directly in clinical decision-making. During and at the conclusion of the course you will have the opportunity to engage a genetic counselor and Sinai’s medical geneticists to follow-up on any potentially clinical significant findings.

You are encouraged to seriously consider what information you would like to know from WGS and what you would prefer not to learn about. It may not be possible for you to avoid learning certain types of information about yourself, and if you feel very strongly that you do not want to learn some types of information that could arise, then you should consider using a publicly available, anonymous genome rather than your own genome data.

You will have the opportunity to change your decision at any time, including after you actually receive your personal genome data.

You are eligible to participate in the course whether you prefer to analyze your own or an anonymous genome.

To reiterate, you do not have to have a blood draw, and do not have to obtain your personal genome data, in order to participate in the course.

**Blood draw**

At the end of your session today, if you do opt to have a blood draw, we will take a sample of your blood for whole genome sequencing. About 40 ml of blood (3 Tablespoons) will be drawn from one of your veins. As soon as your blood is taken, the tubes will be labeled with a sticker that contains a unique “barcode.” No other identifying information will be on the blood specimens. Another sticker with the same barcode will be placed on the back of your WGS information sheet and immediately put in a locked cabinet. The link between your barcode number and your identity will be kept confidential and will only be known by the study staff who are unaffiliated with the Practical Analysis of Your Personal Genome course.

**Analyzing your blood for whole genome sequencing**

We will obtain genetic material (DNA and RNA) from the blood cells at Mount Sinai School of Medicine. Your sample will only be identified by the unique barcode. No personal information (such as name, date of birth, medical record number) will be provided to the laboratory that analyzes your sample or provides the initial annotation of the data. Please note that the WGS test is not clinically approved by New York State and is being done for educational purposes only.

**Returning WGS data to you**

The raw data produced by the sequencing instruments will be processed into FASTQ files by the sequencing core staff according to the core’s standard operating procedures. These FASTQ files will be stored on Minerva, Mt. Sinai’s scientific computing system, in directories only accessible to core’s staff and with filenames containing only the anonymous identifiers described above. The core staff will send pointers to these files to the research coordinator who will forward those pointers to you. If you elect to use an anonymous genome you will be matched to the most similar (in terms of sex and ethnicity) available genome sequence by an informatician not associated with the course. These relevant files will use the same naming scheme as those described above so as to be as indistinguishable as possible from those sequenced for the course. The research coordinator will send pointers to these files to you. In either case, you will copy your files to directories on Minerva to which only you have permission to access.

**Interpretation of WGS data**

All data will be analyzed and interpreted directly by you, not the course instructors or other Sinai clinicians or researchers. Analysis steps will include (but are not limited to) quality assurance and filtering based on sequencing and variant calling quality calling measures; intersection with databases of variants with known disease or pharmacogenomic associations; calculation of disease risk for complex polygenic diseases (e.g., Type 2 Diabetes); mathematical analysis of ancestry; and assessment of variants of unknown significance. The last will incorporate what, if any, amino acid changes may result, measures of novelty and rarity in the population, evolutionary conservation, various inheritance models and prediction of functional effect (i.e. is the variant predicted to be damaging to the protein function). As part of the course, you will be guided through an analysis workflow that will follow Mt. Sinai’s clinical sequencing workflow.

**Storage of WGS data and blood/DNA specimens**

Your blood/DNA specimens and WGS data are being collected for educational purposes only, and will not be retained or banked for any research purposes, other than to provide to you for you to analyze and interpret yourself.

**Blood/DNA sample:** The blood sample will be retained only long enough to complete the DNA sequencing: the blood sample will be destroyed after sequencing is completed.

***WGS data:*** If you opt to receive your WGS data, the FASTQ files (WGS data) will be made available to you on Minerva in a location in which only you can access. The WGS data will not be retained by the Institution or used for any purpose other than to provide to you for educational purposes.

**Optional Genetic Counseling Session to Review Results of WGS**

Should you wish to discuss any aspect of the results you obtain via analysis of your own personal genome data, you will be provided the option of seeing a genetic counselor at MSSM who is unaffiliated with the course (Claire Davis) or a genetic counselor not affiliated with MSSM or the course at Columbia University. Because the WGS data is being performed and interpreted in a non-clinically approved research lab, the genetic counselor might recommend appropriate testing and follow up if there is significant concern about a result. This might include having specific genetic tests (if available) repeated or “confirmed” in a clinically approved laboratory before making any medical decisions. All follow up testing would be performed at your own cost and is not covered by this research and educational project.

**Public Sharing of your genome data**

Some individuals have chosen to make their whole genome data public, so that researchers around the world can learn more about the human genome. You will have the opportunity to share your genomic information publicly, e.g. you may choose to share your data with a National Institutes of Health (NIH) sponsored database, dbGaP. dbGaP was developed by the NIH so that researchers around the world can share genetic information to enhance research. Access to personal information, like whole genome sequencing data, is restricted by the NIH to certain investigators. dbGaP only accepts data that has no personal information or identifiers, so if you choose to submit your whole genome data to dbGaP, you will need to submit it without any information that could potentially identify you (your name, date of birth, etc.).

| YOUR RESPONSIBILITIES IF YOU TAKE PART IN THIS COMPONENT OF THE COURSE: |
| --- |

If you decide to take part in the sequencing component of the course you will be responsible for the following things: attending the information and blood draw session, and completing the blood draw.

| COSTS OR PAYMENTS THAT MAY RESULT FROM PARTICIPATION: |
| --- |

If you agree to take part in the sequencing component of the course, you will not be paid for doing so. The genetic counseling sessions and whole genome sequencing will be provided to you at no cost.

| POSSIBLE BENEFITS: |
| --- |

We cannot and do not guarantee or promise that you will receive any direct benefit from participating in the sequencing component of this course. However, others might potentially benefit from your participation in this component of this course and the associated questionnaire study. Helping us understand the pros and cons of providing students with the option of analyzing their own personal genome data may help students who might choose to enroll in whole genome sequencing courses of this type in the future.

| REASONABLY FORESEEABLE RISKS AND DISCOMFORTS: |
| --- |

There are risks, discomforts, and inconveniences associated with any procedure of this kind. These deserve careful thought. In addition to what is described below, there may be unforeseeable risks that occur as a result of genome sequencing and your interpretation of the data this produces.

**Risks related to coercion:**

One risk of participating in the class is that you will feel coerced into getting your genome sequence data in order to please the course directors, and may feel that if you don’t do these things then you may jeopardize your grades.

You absolutely do not have to get you genome sequenced. Your genome data will be kept completely separate from your name. Your genome sequence data will be accessible only to you. The course directors will never see your genome data, nor know whether or not you chose to receive your own genome data or that of an anonymous, publicly available genome. In addition, this will be a pass-fail class, so there will be no grades allocated on this course.

**Risks related to blood draw:**

The risks of a blood draw include pain, bruising, and the slight possibility of infection at the place where the needle goes in. Some people feel dizzy or may faint during or after a blood draw.

The procedures for minimizing risk during blood drawing include the use of a special lounge chair that helps participants relax during the procedure, the use of a numbing cream, if needed, and the use of individuals trained and certified in drawing blood.

**Risks related to learning genetic information:**

There is a chance that you may learn that you carry a gene mutation that may increase risk for a specific medical condition. Although you will have the option of seeking a referral for medical help or risk management as appropriate this knowledge might be upsetting and may cause anxiety or psychological distress. As described above, some of the conditions you learn about may have treatment or screening options available, while others may not. Some of these conditions may also be potentially stigmatizing. Please think about if you want this information before the data is available. You may learn that a family member is at risk of developing certain medical conditions or diseases. You may also learn that ancestry or parentage is different than previously thought. This may cause some psychological distress and can be upsetting.

**Risks related to privacy:**

Your privacy is very important to us, and we will use many safety measures to protect it. However even with all of these protections, there is the possibility that the genome sequence data derived may, even when presented without other identifying factors, allow you to be re-identified, and therefore we cannot promise anonymity, particularly if you choose to publish or share your genome sequence data with others.

Specific illnesses and known genetic problems may be found by examining DNA. In the future, insurance companies may use this information to determine if someone is able to be insured by their company. If you tell your family doctor that you have had your genome sequenced, or if you tell your doctor about any specific aspects relating to your genome sequence data, this information may then become part of your medical record with this doctor. Insurance companies routinely have access to such records. You will be the sole owner of your genome data, and so there is no risk of your information being released about you or your family to your doctor unless you choose to do so. While your DNA is being sequenced, the investigators will keep your identifying information separate from the DNA. The DNA will be linked to your personal information only through an ID number (“barcode”). We will keep linking information under lock and key and confidential to the extent permitted by law.

There is a small risk that you may face discrimination on the basis of genetic predispositions that are identified through your genome being sequenced. Sometimes patients have been required to furnish information from genetic testing for health insurance, life insurance, and/or a job. A Federal law, the Genetic Information Nondiscrimination Act of 2008 (GINA), generally makes it illegal for health insurance companies, group health plans, and employers with 15 or more employees to discriminate against you based on your genetic information. It is important to note that GINA does not provide protections against discrimination when applying for long term care insurance, disability insurance or life insurance.

There always exists the potential for loss of private information; however, there are procedures in place to minimize this risk.

| OTHER POSSIBLE OPTIONS TO CONSIDER: |
| --- |

You may decide not to have your genome sequenced without any penalty. This choice is totally up to you.

| IN CASE OF INJURY DURING THIS PROCEDURE: |
| --- |

If you believe that you have suffered an injury related to having your genome sequenced as part of this course, you should contact Research Coordinator Micol Zweig (212-659-8503), or one of the two following course directors: Dr Michael Linderman (212-659-8546) or Dr. Saskia Sanderson (212-659-8520).

| ENDING PARTICIPATION IN THE SEQUENCING COMPONENT OF THE COURSE: |
| --- |

You may stop taking part in the sequencing component of the course at any time without any penalty. This will not affect your ability to receive medical care at Mount Sinai or to receive any benefits to which you are otherwise entitled.

If you decide to stop being in the sequencing component of the course, please contact Research Coordinator, Micol Zweig (micol.zweig@mssm.edu).

While your samples are being sequenced, your samples can be withdrawn from the repository and destroyed if you request this from Micol Zweig.

Withdrawal without your consent: The sponsor or the institution may stop your involvement in the sequencing component of this course at any time without your consent. This may be because the course is being stopped, the instructions of the course staff have not been followed, the staff or investigators believe it is in your best interest, or for any other reason. If specimens or data have been stored as part of the project, they too can be destroyed without your consent.

If consent to store the tissue sample is withdrawn at any time prior to it being destroyed as per the project protocol, the repository storing the sample will promptly destroy the sample.

| CONTACT PERSON(S): |
| --- |

#### If you have any questions, concerns, or complaints at any time about this project, or you think the project has hurt you, please contact Micol Zweig at 212-659-8503, Dr Michael Linderman at 212-659-8546, or Dr. Sanderson at 212-659-8520.

| DISCLOSURE OF FINANCIAL INTERESTS: |
| --- |

None.

Signature Block for Capable Adult

| Your signature below documents your permission to take part in the sequencing component of this course. A signed and dated copy will be given to you. | | |
| --- | --- | --- |
| DO NOT SIGN THIS FORM AFTER THIS DATE | 🡪 |  |
|  |  |  |
| Signature of student |  | Date and Time |
|  |  | |
| Printed name of student |

Person Explaining Procedure and Obtaining Consent

|  |  |  |
| --- | --- | --- |
| Signature of person obtaining consent |  | Date and Time |
|  |  |  |
| Printed name of person obtaining consent |  |  |

*If the individual cannot read, a witness is required to observe the consent process and document below:*

| *My signature below documents that the information in the consent document and any other written information was accurately explained to, and apparently understood by, the subject, and that consent was freely given by the subject.* | | |
| --- | --- | --- |
|  |  |  |
| *Signature of witness to consent process* |  | *Date and Time* |
|  |  | |
| *Printed name of person witnessing consent process* |
