# Supplementary material for: Informed decision-making among students analyzing their personal genomes on a whole genome sequencing course: a longitudinal cohort study
Source: Genome Med. 2013 Dec 30;5(12):113. doi: 10.1186/gm518 (PMC3971344; doi:10.1186/gm518)
Supplement: Additional file 10 — Supplementary methods. [file gm518-S10.docx]

**SUPPLEMENTARY METHODS**

**Methods**

The Associate Dean for Undergraduate Medical Education and Curricular Affairs sent out an email advertising the courses in May 2012 to all second, third and fourth year medical students at ISMMS. The medical students were informed that the introductory course was available to second year medical students as a no-credit, and to third or fourth year medical students for elective credit. They were also informed that the advanced course would be open to fourth year medical students who had completed the introductory course, for elective credit. The director of the Masters Program informed the genetic counseling students about the course in-person. For these students, the courses were offered as a required part of their core curriculum. The course was advertised to residents, fellows and PhD students in the Department of Genetics and Genomic Sciences at ISMMS in an email sent out in June 2012 from the Vice Chair of the Department. Faculty members were also informed about the course this way. Interested students could sign up either via an ISMMS website, or by emailing the Vice Chair of the Department. All students who enrolled on the introductory course were eligible to participate in the research study. Twenty students were chosen to enrol on the introductory course.

One week before the first class of the introductory course, a course director emailed the first information sheet about the research study to the students to enable them to read and prepare any questions they might have about it. This 4-page information document informed them that the first part of the study consisted of them answering two questionnaires, one in the current class, and one at the last class on the introductory course; that each questionnaire would take 15-30 minutes to complete; and that risks included those related to privacy (for this first research information sheet see Additional File 7).

When the students arrived for their first class on the “Introductory to Human Genome Sequencing” course (23 July, 2012), they were informed about the study in-person by one of the study investigators, and paper copies of the first research information sheet was distributed to the students. They were given the opportunity to discuss the information and to ask any questions they wished of the study investigators and course directors. The course directors then stepped out of the room so that the students could ask questions of the research coordinator who remained in the room, so that the research coordinator could administer the 8-page paper questionnaire, and so that the directors were not aware of who did or did not complete the questionnaire.

At the end of the last class of the introductory course (8 August, 2012), the research coordinator administered an identical questionnaire, again with course directors having stepped out of the room. Absence of the course directors preserved the privacy of the students, and ensured that student questions and their choice of completing the questionnaire could not influence their performance in the course or the opinions of the course directors regarding the students.

One week before the advanced, “Practical Analysis of Your Personal Genome,” students were emailed a 2-page information sheet about the next part of the research study (for the second research information sheet see Additional File 8). In this information sheet, the students were informed that this part of the study would involve two further 15-30 minute questionnaires.

Upon arriving for the first (mandatory) class of the advanced course (12 September, 2012), students were again given the opportunity to discuss the course and study with the course directors, before the directors stepped out of the room: as previously, the questionnaires were administered by the research coordinator, who also answered any further questions the students had. In this class, students were also informed about the WGS part of the class by the course directors, and informed that they had the option of attending a session to receive further information from the research coordinator and to have the blood draw for WGS if they so chose.

All questionnaires were identified only by a study number; the students’ names and other information that could identify them were not included on the questionnaires. The study number was ‘linked’ to their name in a secure database that was not accessible to the course directors.

Students wishing to use their own personal genomes in the class contacted the research coordinator and made an appointment to learn more about the WGS, ask questions, and have the blood draw. During this session, they were given an 8-page information sheet about the personal WGS as part of the educational course (for this information sheet see Additional File 9). The research coordinator went through the information sheet in detail with them, and gave them the opportunity to ask questions. The students were informed that WGS was not currently a clinically approved test in New York State and was being done for educational purposes only. They were reminded that the introductory course had provided detailed background into the types of information that can be learned from WGS and the limitations thereof, and were reminded of key details, including that a large number of unique variants in their DNA that may be difficult to interpret would likely be identified. The students were informed that their raw data produced by the sequencing instruments would be processed into FASTQ files, that these would be stored on Minerva, ISMMS’s scientific computing system, in directories only accessible to the staff of the ISMMS Genomics Core Facility that generated the data, that they would then copy their files to directories on Minerva to which only they, the students, had permission to access, and that all other copies of their genome data would then be destroyed at that time. They were also informed that the blood sample would be destroyed after sequencing was completed, and that the WGS data would not be retained by the Institution or used for any purpose other than to provide to the students for educational purposes. Risks covered included those related to coercion, the blood draw, learning genetic information, and privacy. It was reiterated to the students that they did not have to have the blood draw, and did not have to obtain their personal genome data, in order to participate in the course.

At this time, the students were also given contact details for a named genetic counselor within ISMMS, and a named genetic counselor outside of ISMMS, for optional pre-WGS genetic counseling. These optional individual genetic counseling sessions were offered to all students at no cost to them before, during and after the advanced course to ensure students had the opportunity to see a genetic counselor to discuss the risks and benefits of receiving such information, and to address any concerns the students may have, in private. They were assured that any information shared and discussed within the genetic counseling sessions would be confidential and private, and would not be shared in an identifiable way with the course instructors or study investigators. Only two students opted to receive genetic counseling, with both seeking counseling from the onsite genetic counselor at the start of the advanced course. One of the two students expressed seeking counseling solely to experience what the genetic counseling was like. None of the students made an appointment to see the genetic counselor offsite. Details for the Student Mental Health Services team at ISMMS were also provided.

**Questionnaire measures**

The major classes of primary variables that were assessed in the questionnaires at timepoints T1, T2 and T3 were: (1) interest in analyzing own genome in the educational setting, including intentions, decision, and discussion of decision with others; (2) decisional conflict (or ‘decision uncertainty’) about analyzing own genome in a classroom, indicative of informed decision making; (3) attitudes towards WGS in the educational setting; (4) attitudes towards WGS in general; and (5) knowledge regarding how to interpret DNA variants associated with disease risk in a clinical setting.

**Interest:** First, we assessed interest in analyzing one’s own genome in a classroom using two items (i.e. survey questions). The first was, *“Would you want to analyze your own genome as part of an advanced whole genome sequencing course?”* (response options: no definitely not / no probably not / yes probably / yes definitely / don’t know / it depends). The second was adapted from the ‘option’ introductory section of the O’Connor’s Decisional Conflict Scale ([O'Connor 1995](#_ENREF_8)): *“At this point, which of the following options would you prefer? Please check one. Option 1: I would like to analyze my own genome as part of an advanced whole genome sequencing course. Option 2: I would not like to analyze my own genome as part of an advanced whole genome sequencing course, and would rather analyze an anonymous donated genome.”* We also assessed ‘perceived utility’ with the item, *“I think analyzing my own genome as part of an advanced whole genome sequencing course would be useful.”* Additionally, we assessed reasons for using their own genomes using 6 items (e.g. *“Understand what a patient may learn/experience”*) and reasons against using their own genomes using 8 items (e.g. *“Results are not reliable”* and *“Unwanted information”*), adapted from Ormond et al ([Ormond, Hudgins et al. 2011](#_ENREF_9)).

**Decisional conflict:** Second, we assessed decisional conflict using an adapted version of the widely used and validated Decisional Conflict Scale (DCS). The original DCS was published by O’Connor ([O'Connor 1995](#_ENREF_8)); we adapted a more recent version of the 16-item DCS published by O’Connor in the 2010 updated DCS User Manual (available from <http://decisionaid.ohri.ca/docs/develop/Tools/DCS_English.pdf>; accessed 8 Nov, 2012). This version of the DCS has 5 subscales: (1) the Informed Subscale, e.g. *“I know which options are available to me”*; (2) the Effective Decision Making (Satisfaction) Subscale, e.g. *“I feel I have made an informed choice”*; (3) the Support Subscale, e.g. *“I am choosing without pressure from others”*; (4) the Values Clarity Subscale, e.g. *“I am clear about which benefits matter most to me”*; and (5) the Uncertainty Subscale, e.g. *“This decision is easy for me to make”,* each with 5 response options from ‘strongly agree’ (which is scored ‘0’) to ‘strongly disagree’ (which is scored ‘4’)*.* For the present study, we used items adapted from this manual (all of the original items were included with the exception of the item *“I am clear about the best choice for me”* from the Uncertainty Subscale; we imputed the missing values for this variable by calculating the mean of the other 15 items for each participant and imputing that mean value for this item). The only other difference between our version of the decisional conflict measures and the version in the User Manual was that we referred to “risks” only instead of “risks and side effects” (e.g. we used *“I know the risks of each option”* instead of *“I know the risks and side effects of each option”*). This modification was made because the term “side effects” does not readily apply to the case of WGS. In accordance with the DCS user manual (see <http://decisionaid.ohri.ca/docs/develop/User_Manuals/UM_Decisional_Conflict.pdf>, accessed 30 January, 2013), total scores for the DCS were calculated by summing the 16 items, dividing by 16, and multiplying by 25, in order to give a total score with a range from 0 to 100. Each DCS subscale score was calculated using a similar approach, in accordance with the DCS user manual. The total DCS has a possible range from 0 (no decisional conflict) to 100 (extremely high decisional conflict). Scores lower than 25 are associated with implementing decisions; scores exceeding 37.5 are associated with decision delay or feeling unsure about implementation.

**Attitudes:** The third class of primary variables we assessed related to attitudes, i.e. perceived benefits (e.g. *“My own results would help me understand genetics concepts better than someone else’s results”*), concerns (e.g. *“I would be concerned that my professors would know who took up the offer of testing and who did not”*), and general views on whole genome sequencing (e.g. *“Physicians have a professional responsibility to help individuals understand the results they receive from whole genome sequencing, even if the physician has not ordered the test.”*), all of which were assessed using items adapted from Ormond et al ([Ormond, Hudgins et al. 2011](#_ENREF_9)). There was also an open-ended section for “Other Comments”.

**Knowledge:** The final class of primary variables that was measured at all four time points measured related to knowledge; this was assessed with a series of questions adapted from Ormond et al ([Ormond, Hudgins et al. 2011](#_ENREF_9)). The knowledge questions asked students to consider three different clinical scenarios in which they had to imagine a patient was presenting them with results from a DTC genetic testing service about their risk of breast cancer, hemochromatosis and macular degeneration respectively. For each scenario, the students were asked *“What is the best way to interpret your patient’s results?”, “What issues impacted your understanding of the case?,* and *“How would you counsel the patient?”.*  Each question was followed by a number of multiple choice options, of which the students were asked to check as many boxes as applied.

The complete surveys used in the course are available as Supplemental Material.
